# The interaction of healthcare service quality and community-based health insurance in Ethiopia

**DOI:** 10.1371/journal.pone.0256132

**Published:** 2021-08-19

**Authors:** Bekele Belayihun Tefera, Mengistu Asnake Kibret, Yordanos B. Molla, Girma Kassie, Aynalem Hailemichael, Tarekegn Abate, Hailu Zelelew, Binyam Fekadu Desta, Elizabeth Futrell, Zewditu Kebede, Gebeyehu Abelti, Subrata Routh, Bamikale Feyisetan, Abdulmumin Saad

**Affiliations:** 1 USAID Transform: Primary Health Care Activity, Pathfinder International, Addis Ababa, Ethiopia; 2 Pathfinder International, Boston, Massachusetts, United States of America; 3 USAID Transform: Primary Health Care Activity, Abt associate, Addis Ababa, Ethiopia; 4 Abt associate, Cambridge, Massachusetts, United States of America; 5 USAID Transform: Primary Health Care Activity, JSI Research & Training Institute, Inc., Addis Ababa, Ethiopia; 6 USAID Ethiopia, Addis Ababa, Ethiopia; 7 USAID Contractor, Global Health Bureau, Office of Population and Reproductive Health, USAID, Washington, DC, United States of America; 8 Global Health Bureau, USAID, Washington, DC, United States of America; University of West London, UNITED KINGDOM

## Abstract

Community-based health insurance (CBHI) as a demand-side intervention is presumed to drive improvements in health services quality, and the quality of health services is an important supple-side factor in motivating CBHI enrollment and retention. There is, however, limited evidence on this interaction. This study examined the interaction between quality of health services and CBHI enrollment and renewal. A mixed-method comparative study was conducted in four agrarian regions of Ethiopia. The study followed the Donabedian model to compare quality of health services in health centers located in woredas/districts that implemented CBHI with those that did not. Data was collected through facility assessments, client-exit interviews, and key informant interviews. In addition to manual thematic analysis of qualitative data, quantitative descriptive and inferential analyses were done using SPSS vs 25. The process related (composite index including provider-client interpersonal communication) and outcome related (client satisfaction) measures of service quality in CBHI woreda/districts differed significantly from non-CBHI woredas/districts, but there were no significant differences in overall measures of structural quality between the two. The study found better diagnostic test capacity, availability of tracer drugs, provider interpersonal communication, and service quality standards in CBHI woredas. A higher proportion of clients at CBHI health centers gave high ratings of overall satisfaction with services. Individual and household factors including family size, age, household health care-related expenditures, and educational status, played a more significant role in CBHI enrollment and renewal decisions than health service quality. Key-informants reported in interviews that participation in the scheme increased accountability of health facilities in CBHI woredas/districts, because they promised to provide quality services using the CBHI premium collected at the beginning of the year from all enrolled households. This study indicates a need for follow-up research to understand the nuanced linkages between quality of care and CBHI enrollment.

## Introduction

Timely access to health care can minimize the impact of ill health. Health insurance can reduce the cost of accessing health services and lessen the impact of ill health on the household’s ability to earn income [1,2]. Many low-income countries have introduced revenue-supported health insurance for urban informal workers and rural residents. With a mix of government co-funding, premiums, and registration fees, households can ameliorate the impact of illness by sharing risk and spreading out the costs of health care over time. Community-based health insurance (CBHI), often administered by communities or local governments, elicits voluntary enrollment for a fee for a period of one year [3,4]. In return, enrollees receive a specified set of services with no out-of-pocket co-payment at the point of care. CBHI has been promoted widely in low- and middle-income countries [5]. However, enrollment into CBHI or other types of insurance has historically been low in sub-Saharan Africa [6].

With the aim of ensuring access to quality health care services by all citizens without financial burden, the Ethiopian government established the Health Insurance Agency [7]. In 2011, the agency began piloting a CBHI scheme in 13 woredas in four of its largest agrarian regions (Amhara; Oromia; the Southern Nations, Nationalities, and Peoples’ Region [SNNPR]; and Tigray) [8]. Prompted by the pilot’s success and viability, the government of Ethiopia scaled CBHI up to 512 woredas in six regions, expanding to Benishangul-Gumuz, Harari, and two city administrations (Addis Ababa and Dire Dawa) [8,9]. As of 2019/20, CBHI schemes are functional as part of a phased national rollout plan, in 700 woredas and cities—covering some 32.2 million people or almost a third of Ethiopia’s population [10]. The voluntary CBHI scheme is available to households in rural, semi-urban, and urban areas who are not employed within the formal sectors of the economy. The scheme charges member households a nominal one-time registration fee along with a yearly enrollment/renewal premium. The annual premium is between USD 7 and 8 per rural household and USD 10 and 14 per urban household. A general subsidy from the federal government matches 10% of the CBHI enrollment proceeds. Also, local governments at the woreda and regional level allocate a certain amount of budget as targeted subsidy each year to cover the free enrollment of select poor and indigent households unable to pay for CBHI membership [11]. CBHI covers basic costs for inpatient and outpatient services at public health facilities within a member’s catchment area.

Enrollment in CBHI reflects a financial investment in the health system by individual citizens. At the same time, increased demand for quality services has the potential to lower average quality if limited resources are strained. Since 1998, the Ethiopia Ministry of Health has invested and given health facilities autonomy to use the revenue they collect from user fees and other sources to improve access to and quality of healthcare service. Studies in different countries, including Ethiopia, focus on factors that affect CBHI enrollment and renewal and the role of CBHI on service quality [1,12–18]. Findings showed that long waiting times, poor patient-provider interactions, unavailability of diagnostics and essential drugs, unfavorable client perception of health care quality, and low client-service satisfaction were associated with high CBHI scheme dropout rates. This indicates that quality of healthcare affects decisions about whether to reenroll in CBHI [19–22]. However, service quality was not shown to be the reason for CBHI drop-out rates in pilot areas [12]. Enrollment rates were also affected by timing, the payment modality involved in enrolling into the insurance, trust in scheme management, and distance to health facilities. Acceptance of modern medicine or attitude toward planning for future illnesses might also affect enrollment [19,20,23,24]. Quality of health services is critical for member satisfaction and sustainability of the CBHI scheme. As CBHI is scaled up and more time passes, the role of quality of health services in CBHI enrollment in Ethiopia might become more evident.

The Ethiopian Hospital and Primary Health Care Alliance for Quality guidelines steer efforts to enhance the health care delivery structure and process and improve the client experience of care. By aiming to increase the availability of supplies, use new tools, apply innovations, strengthen providers skills, and improve work environments, implementation of CBHI in a woreda has the potential to ensure a threshold level of quality of healthcare service. CBHI provides financial protection to its members and increases health care seeking behavior and health service utilization [25,26]. This, in turn, creates pressure on health facilities and providers, and may affect the quality of healthcare service at the health centers, but there is limited evidence on the interaction between CBHI and quality of health services. This study examined the interaction between quality of healthcare service and CBHI uptake. The following research questions were explored: (i) Has the introduction and implementation of CBHI improved quality of healthcare services? and (ii) Does quality of healthcare affect enrollment in and renewal of CBHI?. The study presents quality of healthcare services through the Donabedian model, which posits that the presence of structural quality facilitates process quality, which leads to outcome quality based on client experience and satisfaction [27]. This model was adopted because it explores all the three elements of quality and postulates that the CBHI implementation mechanism itself and the related enhanced revenue generation would improve quality of health care service; enhanced quality of health care service or perceived quality itself would generate greater enrollment into CBHI.

## Methods

### Study area and period

Since 2017, USAID Transform: Primary Health Care project, in collaboration with the Ministry of Health, has supported continuous improvement of quality of healthcare services in Ethiopia. Benefiting nearly 53 million people, Transform strengthens the management and performance of Ethiopia’s national health system by improving quality of service delivery across the continuum of primary health care, improving household and community health practices and health care-seeking behaviors, and strengthening program learning to impact policy and activities related to the prevention of child and maternal deaths. The study was conducted in selected health centers located within, USAID Transform: Primary Health Care implementation regions and focused on interaction between CBHI enrollment and healthcare service quality. Data was collected from August to September 2019.

### Study design

An embedded mixed-method comparative study was conducted in four agrarian regions of Ethiopia (Amhara, Oromia, SNNPR, and Tigray) to provide information on quality of healthcare services in woredas that implemented CBHI and those that did not. The study was guided by the Donabedian model.

### Sampling and sample size determination

As there is no pre-determined estimate of level of differences to base our assumptions for estimating sample size, standard sample size formula could not be used to calculate sample. Woredas were selected from the four regions where USAID Transform: Primary Health Care is implemented. The sampling frame included woredas that have implemented CBHI for at least 24 months and non-CBHI woredas that are in USAID Transform: Primary Health Care areas. Within the CBHI and non-CBHI groups, woredas with health centers that initiated a quality improvement (QI) intervention with the project’s support and woredas with health centers that have not yet initiated a QI intervention were included. Two woredas from each category were randomly selected from Amhara, Oromia, and SNNP, and a single woreda was randomly selected from Tigray for each of the four categories. A total of 28 woredas were selected from the four regions.

Two health centers were randomly selected from each of the 28 woredas for a total of 56 health centers. Exit interviews were conducted among participants who used in the selected health center for any health services. Participant selection was completed through consecutive sampling method until an adequate sample from each health center was obtained. The plan for exit interviews was to include one participant from a health center in non-CBHI woreda for every three-participants interviewed from a health center located in CBHI woreda. For each health center in a woreda that was not implementing CBHI (non-CBHI woreda), five participants were interviewed while 15 were interviewed from health centers located in CBHI woredas. This provided a total of 140 interviews from participants in non-CBHI woredas and 420 interviews with participants in CBHI woredas (which included 224 currently CBHI enrolled, 112 who dropped out of CBHI and 84 who never enrolled participants) for a total of 560 study participates.

The qualitative key informant interviews were conducted with people who had experience with the interaction between CBHI and quality of health services. With the same sampling techniques used in quantitative data collection, 28 key informant interviews were conducted: four with regional officials, four with zonal officials, four with woreda CBHI officials or CBHI management team members (in CBHI woredas), eight with woreda office heads (four from CBHI woredas and four from non-CBHI woredas), and eight with health center heads in each of the study’s regions.

### Data-collection process and instruments

**Fig 1:**
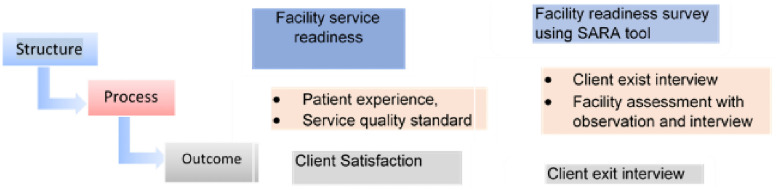
Quality of care measurement domains and data sources.

The assessment used three data collection methods (Fig 1). Structural-quality measures were investigated through a facility survey with a standard tool. Process-quality measures were investigated based on exit interviews with participants about their perception of provider adherence to quality standards and procedures, as well as key informant interviews at health facilities, woreda, zonal, and regional health bureaus. Outcome-quality measures were explored based on client satisfaction as they exited the health facility.

#### Participants exit interview

Exit interviews were conducted using a structured questionnaire translated into local languages with 560 participants who visited health centers selected for the study on the day of data collection. Along with participant demographic information, the exit interviews elicited participants’ views on health care services received during the visit preceding the interview, previous experience in the same health center, and attitudes toward CBHI in general. In addition to individual characteristics (such as age, sex, marital status, education) and out-of-pocket expenditure during the facility visit on the day of the interview, eight indicators were used to measure client service satisfaction, comparing clients from CBHI woredas with their counterparts. These indicators were: overall quality of service, availability of medicines and supplies, availability of diagnostic services, cleanliness of facility, wait time between arrival and service, wait time between services, friendliness of staff, and availability of private counseling services. In addition to client service satisfaction, the exit interview included tracer indicators that were used to develop composite measures of interpersonal relations (4 indicators) and client perception about provider adherence to proper procedure (10 Likert scale indicators). The exit interview instrument was adapted from the evaluation tool used for the CBHI pilot in Ethiopia [28].

#### Facility survey

Structure quality was assessed in 56 health facilities using a standard facility assessment tool focused on an inventory of availability and readiness of basic health facility infrastructure: basic amenities (6 tracer items), basic equipment (6 tracer items), diagnostic capacity (7 tracer items), essential medicines (25 tracer items), and standard precautions for infection prevention (9 tracer items). In addition to facility surveys, interviews were conducted with facility heads and supplemented with observation of facility adherence to service quality standards. Observation of service quality standards included 18 tracer indicators on the facility’s QI structure, capacity to implement QI, providers’ ability to provide patient-centered care, patient-safety practices, availability of quality monitoring and sharing processes, and practices for QI learning. The facility survey instrument and analysis methodology were adopted from WHO’s SARA tool [29]. The facility service quality standard assessment tool was adopted from the national standards used for facility quality inventory [30].

#### Key informant interviews

A qualitative assessment to understand the design, operation, and implementation of CBHI at different levels of the health system was conducted with 28 key informants who were heads of regional health bureaus, zonal offices, woreda health offices, health centers, as well as woreda level CBHI focal persons. The questionnaires were prepared in English, translated into the local languages (Tigrigna, Amharic, and Oromifa), and translated back into English to check consistency. Twenty data collectors who were fluent in the local language (five for each region) were selected for data collection and four supervisors with experience in CBHI, and service quality integration were selected for supervision. Training, consisting of mock interviews and practical exercises for both data collectors and supervisors, was conducted over five days in July 2019. The questionnaires were pretested and refined to ensure they were clear and could be understood by both the data collectors and respondents.

### Data management and analysis

The research team assessed the quality, accuracy, and completeness of the collected data using range plausibility and cross-validation checks. The data was collected using local languages and back translated in English before analysis. The quantitative data was entered into EPI-Data vs 3.02 for Windows and exported into SPSS vs 25 for further analysis. Data analysis consisted of descriptive statistics and comparisons across woredas as defined by CBHI status. Composite indicators (summary measures) were analyzed and compared. A two-level hierarchical linear model was performed to study the relative strength of structure, process, and individual characteristics in predicting client satisfaction with quality of services. Similarly, logistic regression was used to determine the predictors of CBHI enrollment. A 95% level of significance was considered for variables found to have significant association (p-value ≤0.05) with the outcome variable.

#### Qualitative data analysis

The key informant responses were audio-recorded, transcribed verbatim in local languages (Amharic, Oromifa and Tigrigna) and translated into English. Thematic analysis was used to analyze the data in three phases: preparation, team organization and reporting the summary result in each team. The first phase of the analysis started with careful reading of the data multiple times to become immersed in and familiar with the data. In the organization phase, each transcript was read carefully by the first author who highlighted the theme text (words or phrases) that appeared to describe the phenomenon under the study (use of CBHI, quality service, interaction). The highlighted theme text was openly and manually coded with descriptors. The third author read the data to confirm the descriptive codes. These codes were revised, and the codes that emerged from the revision were jointly reviewed before they were integrated into the analysis. The other authors collaborated with the first and third author to review, discuss and agree on the final code categories. The final analysis was summarized manually based on agreed emerging themes.

### Ethical considerations

Ethical approval was obtained from Amhara, Oromia, SNNPR and Tigray Regional public health research institute institutional review board (IRB) committees, and permission letters were secured from the sampled health facility head. Each respondent gave informed verbal/oral consent after being told the purpose, risks, benefit, confidentiality, voluntary nature of participation, whom and how to contact principal investigators and procedures of the study in the presence of head of the facility. All respondent identifiers were kept confidential, and data were anonymized.

## Results

Table 1 describes basic data by woreda insurance and personal insurance status within the woredas where CBHI was introduced. Woredas with insurance show higher age; it is likely that an older population is less educated and may also have a larger family. The differences in health expenditure across the woredas indicate that insurance has reduced health expenditure in the past year at point of care.

**Table 1:** Sample characteristics by woreda insurance status.

Categories	CBHI N = 415	Non-CBHI N = 141	All N = 556
Age	38.3	33	37
Some education (Proportion)	0.51	0.64	0.54
Income yearly, Birr^2^	16600	16600	16600
Family size	5.21	4.8	5.11
Health Exp Last year, Birr^2^	820	1204	925
Health Care Exp, this visit^2^	65	39	46
Distance to HF, minutes^2^	42	42	42

^1^Reporting at p-value < 0.05 unless otherwise stated.

^2^Some figures are rounded off and there is consistent reporting rounding off for some of these figures.

Tendencies to report to nearest 5 and 0 are common in many surveys.

### Has the introduction and implementation of CBHI improved quality of health services?

Structural quality for health centers located in CBHI woredas showed general service-readiness index mean score (a composite summary measure combining information from observation of five general service-readiness domains: basic amenities, standard precautions, basic equipment, diagnostics, and essential medicines) of 0.62. This implies facilities’ readiness to provide 62% of general health services (Table 2). There was no statistically significant difference between overall structural quality in CBHI and non-CBHI health centers based on the general service-readiness index (mean difference = -0.002, p<0.9). However, the average diagnostic test capacity and availability of tracer drugs was better in CBHI woredas and availability of basic equipment was better in non-CBHI woredas.`

**Table 2:** Descriptive statistics for service-readiness and general-process-score index.

Aggregated index	CBHI		Non-CBHI		Mean difference	Sign (2-tailed)
	Mean	SD	Mean	SD		
**Service-readiness**						
Basic equipment	0.78	0.17	0.81	0.16	-0.03	<0.048**
Diagnostic test capacity	0.46	0.34	0.43	0.34	0.03	<0.510
Basic amenities	0.62	0.16	0.63	0.17	-0.01	<0.457
Tracer drug	0.47	0.35	0.43	0.31	0.04	<0.220
Standard precautions for infection prevention	0.77	0.19	0.79	0.19	-0.02	<0.210
**Process-quality**						
Perceived provider followed proper procedure	0.50	0.25	0.50	0.27	0.00	<0.888
Perceived interpersonal relations	4.67	0.58	4.55	0.67	0.11	<0.077*
Observed availability of quality standard	0.61	0.23	0.54	0.26	0.06	<0.011**
**Overall service readiness index**	0.62	0.17	0.62	0.17	-0.002	<0.904
**Overall process index**	1.92	0.23	1.87	0.31	0.05	<0.065*

N = 556 Individual within 56 health facilities.

Significance levels for P values

*** p<0.01,

** p<0.05,

* p<0.1.

The general process score is a composite index of observed availability of service-quality standards, clients’ perception of providers adherence to proper procedures, and clients’ perception of interpersonal relations. This score showed differences between CBHI and non-CBHI woredas. Availability of service quality standards was better in CBHI woredas with a statistically significant difference between health centers located in CBHI and non-CBHI woredas (mean difference = 0.06, p<0.01). Client perception about interpersonal relationships between clients and service providers was also slightly better in CBHI woredas, but the difference was not statistically significant (mean difference = 0.11, P<0.08). The mean value of the general service process index for health centers located in CBHI woredas was perceived to follow proper service processes 92% of the time, while health centers in non-CBHI woredas were perceived to follow proper service processes 87% of the time. However, this difference was not a statistically significant (mean difference = 0.05, p<0.06).

Clients were asked to rate their level of satisfaction from very satisfied, to neutral and to very dissatisfied, based on their experience of service received (Table 3). More than 92% of respondents from health centers located in CBHI woredas were either satisfied or very satisfied with overall service quality. A higher proportion of clients of health centers in CBHI woredas gave high ratings of overall satisfaction (very satisfied/satisfied) than those in non-CBHI woredas (mean difference = 0.36, P <0.001).

**Table 3:** Patient satisfaction with service received (%).

Quality measures	CBHI woreda respondents			Non-CBHI woreda respondents		
	Very satisfied/satisfied	Neutral	Dissatisfied/very dissatisfied	Very satisfied/satisfied	Neutral	Dissatisfied/very dissatisfied
**Satisfaction indicator**						
Overall quality of service	92.4	5.1	2.6	84.8	7.2	7.9
Availability of drugs/medical supplies	89.2	3.9	6.8	79.3	9.3	11.4
Availability of diagnostic service	92.3	3.6	4.1	82.1	7.5	10.4
Cleanliness of facility	92.2	5.1	2.7	89.9	5	5
Short wait time (from the time of arrival in the health facility to seeing the health professional)	89.1	2.9	8	82.1	5	12.8
Short wait time between services (between consultation and diagnostics)	87.7	2.4	9.9	84.3	2.9	12.8
Friendliness of staff	92.7	4.1	3.1	87.1	5.7	7.1
Availability of private counseling service	94.7	1.9	3.4	87.1	5.7	7.1
**Overall satisfaction of clients (mean score values)**	4.25			3.89		
	Mean difference = 0.35; P<0.000***					

N = 556 Individual within 56 health facilities.

Significance levels for P values

*** p<0.01,

** p<0.05,

* p<0.1.

Following the comparative analysis, a two-level hierarchical linear model was used to analyze predictors of overall satisfaction of respondents living in CBHI and non-CBHI woredas. There was a significant heterogeneity among health centers regarding satisfaction with perceived service quality (Table 4). The intraclass correlation coefficient (ICC) in the null model for overall client satisfaction was 37.9%. This means that 38% of the variation in overall client satisfaction between the health centers is due to the difference between the health centers (cluster variation). The fixed estimate, that is estimated overall average satisfaction score across all health centers and client (individual), is 4.1 and is statistically significant. The fixed-effect model indicates that among the individual-level characteristics, client perception of interpersonal relations, perception of provider adherence to proper procedures, and client’s spending on non-medical costs were significantly associated with overall average client satisfaction (p<0.05). No facility-level variables significantly contributed to overall average client satisfaction with service quality. Therefore, predictors of client satisfaction were related to their perception of service received.

**Table 4:** Predictors of overall client satisfaction with quality of health service received.

Characteristics	Coefficient	95%CI	P-values
**Individual level**			
Age	-0.001	(-.003,.002)	<0.730
Sex	0.01	(-.067,.086)	<0.802
Marital status	-0.07	(-.156,.0148)	<0.105
CBHI enrollment status	0.06	(-.028,.145)	<0.191
Spent non-medical expenses during facility visit	-0.001	(-.001, -.000)	<0.021**
Educational status			
Illiterate ^(R)^			
Primary (1–6)	-0.01	(-.097,.082)	<0.86
Secondary (7+)	-0.1	(-.17,.023)	<0.14
Perception of interpersonal relations	0.46	(.392,.545)	<0.000***
Perception of proper procedures followed by providers	0.47	(.314,.627)	<0.000***
**Facility level**			
Observed availability of service quality standard	0.36	(-.764,.036)	<0.075*
Basic equipment	0.03	(-.655,.705)	<0.942
Basic amenities	-0.20	(-.794,.396)	<0.512
Tracer drug	0.21	(-.403,.824)	<0.501
Diagnostic test	-0.19	(-.734,.357)	<0.499
Standard precautions infection prevention	0.05	(-.446,.548)	<0.841

N = 556 Individual within 56 health facilities.

Significance levels for P values

*** p<0.01,

** p<0.05,

* p<0.1.

### Does quality of care affect enrollment in and renewal of CBHI?

Of the total respondents in the CBHI woredas, 81% were ever enrolled as a CBHI member. Of these, 69% were currently enrolled, and 31% did not renew. More than 79% of respondents in both groups said they enrolled to protect themselves from financial shortfalls when seeking care, and 46% of respondents said they enrolled because the premium was lower than the out-of-pocket payments for user-fees and other associated costs they and their household members incurred every time they sought health care (Table 5). Of the participants currently enrolled in CBHI scheme, 36% named frequent illness as their reason for enrollment. The decision to join a CBHI scheme is made on voluntary basis by each household, nearly 6% of respondents reported pressure to enroll by kebele administration (CBHI officials) during the enrollment process.

**Table 5:** Reasons for enrolling, discontinuing or not enrolling in CBHI scheme (%) (multiple responses).

Reasons for Enrolling or not Enrolling	Currently Enrolled (N = 223), n (%)	Previously Enrolled (N = 99), n (%)	Never Enrolled (N = 77), n (%)
***Reasons for Enrolling in CBHI***			
Frequent illness	80 (36)	10 (10)	
Financial protection	180 (81)	77 (78)	
Fee waived	12 (5)	4 (4)	
Low premium fee	106 (48)	42 (42)	
Pressure from officials	14(6)	13 (13)	
***Reasons for not Enrolling or Discontinuing***			
Infrequent illness		12 (12)	4 (5)
Affordability		21 (21)	17 (22)
Lack of knowledge about either how to enroll or renew		23 (23)	33 (43)
Limited-service availability		7 (7)	14 (18)
Low service quality		11 (11)	4 (5)
CBHI staff not trusted		3 (3)	2 (3)
Long waiting time		4 (4)	4 (5)
Wanted to wait to confirm the benefit from other members		18 (18)	13 (17)17 (22)
Others			

Financial protection and low premiums were the main reasons for CBHI enrollment and renewal. Among those never enrolled (n = 77), lack of understanding of the enrollment process (43%) and affordability (22%) played a prominent role, and some stated they wanted to confirm the benefits of the scheme with others (17%). Similarly, among members who did not renew (n = 99), 23% stated they did not have adequate knowledge about membership renewal, and 21% said that the enrollment fee was not affordable at the time of the renewal period and 11% declined to renew due to low service quality of available health care.

The logistic regression results showed that participant age, household family size, and high health care spending played an important role in CBHI enrollment and renewal. The findings determined a 5% increase in the odds of CBHI enrollment for a one unit increase in respondent age (AOR = 1.05, 95% CI: 1.03–1.07) indicating an increase in CBHI enrollment as the age of the client increases. Household family size was positively associated with enrollment: households with more than one member were 1.3 times as likely to enroll as single-member households (AOR = 1.27, 95% CI: 1.09–1.46). Educational status was significantly associated with renewal: respondents with a secondary level of education and above were less likely to drop out of the CBHI scheme (AOR = 0.89, 95% CI: 0.81–0.98). From a statistical point of view, our regression analysis failed to establish a significant association of service quality indicators (structural, process, and outcome quality) with CBHI enrollment and renewal (Table 6).

**Table 6:** Determinants of CBHI enrollment and renewal.

Indicators	Enrollment		Renewal	
	AOR	95% C. I	AOR	95% C. I
Age	1.05	(1.03–1.07) ***	0.96	(0.93–0.98) ***
Sex (female/male)	1.18	(0.66–2.10)	0.60	(0.29–1.22)
Marital status (unmarried/married)	0.61	(0.31–1.19)	1.28	(0.55–2.99)
Educational level	1.06	(0.98–1.14)	0.89	(0.81–0.98) **
Spent of non-medical cost during facility visits ^1^	0.97	(0.96–0.97) ***	1.04	(1.02–1.05) ***
Family size	1.27	(1.09–1.46) **	0.83	(0.69–0.99) **
Service satisfaction	1.15	(0.62–2.16)	0.85	(0.38–1.86)
Structural-quality index	0.77	(0.07–8.61)	1.38	(0.07–25.66)
Process-quality index	0.62	(0.23–1.63)	1.79	(0.51–6.27)
Constant	0.52		1.01	

Significance levels for P values

*** p<0.01,

** p<0.05,

*p<0.1.

^1^Non-medical cost is a composite cost that includes transport, lodging & food.

### Qualitative findings

Factors influencing CBHI enrollment and delivering quality service are categorized by three themes (service availability and quality, right to access service). All key informant interview respondents said health service quality generally has improved in both CBHI and non-CBHI woredas recently. Clients in CBHI woredas are more likely to demand quality service than those in non-CBHI woredas who are paying out-of-pocket for health care services. This is because CBHI woredas promise to provide quality services using the CBHI premium that is collected at the beginning of the year from all enrolled households regardless of pre-existing health conditions. Their demands include the right to be served on time, the right to access basic drugs and laboratory services within a single facility, and the right to be treated respectfully by providers.

*When we ask patients to buy drugs from a private pharmacy*, *CBHI members started to ask a question like*, “*who is going to pay us*?”, *and* “*who will reimburse our expenses*?”. *They say it is their right to get full service from here [health facility] as they were promised*. *They demand all services to be available at the facility*.

–CBHI health center head

Key informants reported that these client expectations drove CBHI health facilities to work towards fulfilling the minimum quality requirement promised during CBHI enrollment.

One zonal CBHI focal person shared:

*We had a challenge of renewing memberships and enrolling potential new members*. *This was because of the weakness in the service provision*. *Lack of adequate drug supply and poor service delivery had contributed*.

According to the key informant interviews (with z*onal and woreda CBHI focal persons*), drug- and diagnostic-supply shortages in CBHI health facilities have forced providers to refer patients to private facilities for certain services. While alternatives are limited in Ethiopia, drug purchases, for example, can be made in the private sector. The clients were offered reimbursement for the costs they incurred in the private facilities, but because clients did not expect these referrals, these incidents have led to misunderstandings and client dissatisfaction. The study findings showed a shortage of drugs and supplies received from Ethiopian pharmaceuticals supply agency (EPSA) in both CBHI and non-CBHI facilities. However, facilities in CBHI woredas have the advantage of accessing financing from the CBHI scheme to procure drugs and supplies. As another interview participant (woreda CBHI focal person) mentioned, “*In addition to the drugs supplied through EPSA*, *the facilities’ boards have recommended to buy additional drugs from private suppliers and make it available for patients*. *We are trying our best to do that*.”

In summary, comparing quality of health services across health centers in CBHI and non-CBHI woredas, our findings revealed no significant differences at the structural level—for example, in facility-level preparedness to treat illness. But upon examination of specific indicator domains—for example, availability of diagnostics test and tracer drugs, we found minor differences. This and the fact that our exit interviews indicated that clients visiting health centers in both CBHI and non-CBHI woredas reported no structural issues related to quality of health service implies that the health centers are comparable in their overall preparedness to treat illness, but availability of replenishable items such as drugs and diagnostic tests was slightly better in CBHI woredas.

## Discussion

There were differences in process- and outcome-quality indicators across health centers in CBHI and non-CBHI woredas. Provider-client interactions and perceived adherence to service quality standards appeared to influence client satisfaction more than any other factor. Respondents who were already engaged with the health centers might view facility care more positively than the general population. This type of finding has been noted elsewhere [23]. Subjective measures like the Likert scale might yield more optimistic values than those respondents report if probed. A study conducted in Burkina Faso found that, when probed, households gave less favorable answers to questions regarding satisfaction with health insurance than they did initially [31]. It is also possible that those who chose not to renew their CBHI enrollment or who never enrolled participated in the exit interviews to a lesser extent, as they might live further away from health centers. We should expect lower values than those reported for the current study, but we have no reason to think that values in one set of regions were more or less inflated than any other set of regions. Although nearly all respondents reported high satisfaction, clients of health centers in CBHI woredas ranked their satisfaction with services higher than clients of health centers in non-CBHI woredas. Clients in non-CBHI woredas reported lower availability of essential drugs, and when drug supply at the health center was low, more people reported purchasing drugs from private facilities. This phenomenon was not observed in the CBHI areas, indicating that it might be easier for clients in health centers in CBHI woredas to acquire drugs through non-payment.

Individual and household factors played a role in CBHI enrollment and renewal decisions than did quality of health service. Educational status, family size, and age were associated with insurance uptake. These individual factors raise a concern for equity in access to quality health services. Lower levels of education among those who did not renew might indicate the difficulty of reaching these clients with tailored information and education campaigns. The non-CBHI members in CBHI woredas demonstrated lesser understanding of insurance, signaling the need for more effective insurance education activities in the CBHI woredas. Household family size was positively associated with enrollment indicating those with more than one family member are motivated to enroll. The analysis indicated that older people are likely to be ill more frequently and therefore more inclined to enroll in insurance. The insured reported more illness, although this question did not elicit consistent answers from the uninsured. An ever-present problem with voluntary enrollment into insurance is adverse selection: those with higher risk of illness and more in need of health care are the first to enroll. The requirement to launch CBHI in woredas where half of the households in the woreda are willing to enroll can facilitate risk spread among the woreda population at large, strengthening the viability of health insurance and preventing strain on the quality of health service.

Among the primary purposes of health insurance is financing health care at the population level to alleviate the burden of expensive care incurred in a short period of time by individual households. A few insured patients had borrowed large sums of money in previous year before joining the scheme to finance health care costs. This suggests some degree of adverse selection, indicating incomplete risk sharing, as those who enrolled might have higher risk. Adverse selection can be hard to avoid when insurance enrollment is voluntary. Insured participants in this study borrowed less in general and paid much lower prices for drugs to buy from private pharmacies, a common health-care expense. Although there is some payment for care among the insured, it is relatively small. This finding is consistent with Mebratie and colleagues’ research findings, which showed a relationship between non-medical-related costs and enrollment status [12]. Respondents who spent more on these costs were less likely to enroll in or renew CBHI, because the insurance covers only medical treatment and not non-medical expenses such as transport, lodging, or food. Choosing to enroll in insurance is a complicated decision, and quality of health service is only one factor that influences this choice [12,23]. The descriptive and qualitative analysis revealed that quality of health care which includes availability of essential drugs is critical for enrollment and renewal of in the CBHI scheme. However, statistical analysis of the quantitative data showed that structural and process quality indices, and outcome proxy indicators showed no significant influence on CBHI enrollment or renewal decisions. Therefore, quality has not been the deciding factor in uptake of insurance. These results are consistent with other study findings [23]. However, more clients in CBHI facilities expressed satisfaction with the quality of services they received compared to their counterparts. The government has a universal responsibility to deliver an acceptable level of quality health care services in all facilities, whether CBHI or non-CBHI.

This study highlighted important findings to support the interaction between CBHI and quality of healthcare service. However, the data was not conclusive on factors that affect insurance uptake, exposing limitations of the study that also recognize the complexity of linking insurance with quality of health care. First, to show the linkages between facility- and individual-level quality scores and CBHI status, the facility-level was disaggregated into individual-level (client) data. There may be an atomistic fallacy. Second, poor quality of care might discourage people from using health centers altogether. This study explored health center users’ perceptions through exit interviews. The users had already made the choice to visit health centers, which indicates an inclination to value health center care already. Non-CBHI facility users may see a one-time payment as more affordable than the yearly CBHI fee. It is possible that quality of care is a small factor in the enrollment decisions of those already attending health centers and paying for services.

## Conclusion

General structural quality of service was not statistically different between health centers in CBHI and non-CBHI woredas. However, availability of diagnostic test capacity and tracer drugs was slightly better in CBHI woredas than their non-CBHI counterparts. Process- and outcome-related measures of service quality based on clients experience in CBHI woredas scored better than and showed a statistically significant difference from non-CBHI woredas. The quantitative results were supported by key informant interview analysis: CBHI served to improve accountability of health facilities as well as critical aspects of service delivery (like drug availability), hence clients in CBHI facilities were satisfied. Regarding effects of service quality on CBHI enrollment or renewal, only within the woredas where insurance is being implemented and community members would have the choice of enrolling into insurance; showed individual and household (education, family size, age, and health care expenditure,) factors played a significant role in CBHI enrollment and renewal decisions.

The findings revealed that perceived quality of health care is essential for enrollment and renewal. However, health service quality was not shown to have a significant impact on CBHI enrollment or renewal in the statistical analysis of the quantitative data. An important point to note in this respect is ‘clients’ perception of quality, which is based on their experience of care and can differ from observed facility readiness and service availability or process of care provided according to standards and guidelines. Follow-up research is needed for a more nuanced look at the link between service quality and enrollment. Ease of enrollment is an important factor in increasing and retaining CBHI membership. Our findings indicate that households that did not renew CBHI lived further away from health centers, and some might have lacked information about the renewal process. Therefore, CBHI enrollment messages should address existing inequities and reach inaccessible households with tailored messages. Future education campaigns should consider emphasizing that future health risks are likely to be uniform across households and might not be related to the past incidences of ill health. Because age also seems to be a positive factor in enrollment, the program might want to consider offering young families a lower rate or making universal enrollment mandatory.

## Supporting information

S1 Data Study data. (SAV)

S1 File Analysis. (PDF)

S2 File Interview guide. (PDF)

S3 File Survey tools. (PDF)

## References

[1] Acharya A., Vellakkal S., Taylor F., Masset E., Satija A., Burke M., et al. (2013). The Impact of Health Insurance Schemes for the Informal Sector in Low- and Middle-Income Countries: A Systematic Review. *The World Bank Research Observer*, 28(2), 236–266.

[2] Erlangga D., Ali S., & Bloor K. (2019). The impact of public health insurance on healthcare utilization in Indonesia: Evidence from panel data. *International Journal of Public Health*, 64(4), 603–613. doi: 10.1007/s00038-019-01215-2 30737522 PMC6517357

[3] Preker, A., Carrin, G., Dror, D., Jakab, M., Hsiao, W., & Arhin-Tenkorang, D. (2004). Rich-poor differences in health care financing. WHO; http://www.who.int/health_financing/documents/cov-rich-poor-diff/en/. PMC2567719 11953793

[4] Preker A, & Carrin G. (Eds.) (2004). Health financing for poor people: Resource mobilization and risk sharing (pp. 3–52). Washington, DC: World Bank; *vailable at ssrn*: https://ssrn.com/abstract=1026850.

[5] Dror D.M., & Jacquier C. (1999). Micro-insurance: Extending health insurance to the excluded. *International Social Security Review*, 52(1), 71–97.

[6] Appiah B. (2012). Universal health coverage still rare in Africa. *Canadian Medical Association Journal*, 184(2), E125–E126. doi: 10.1503/cmaj.109-4052 22231678 PMC3273529

[7] Kiros, M. (2019). CBHI implementation in Ethiopia. Presentation paper. 21st Annual Review Meeting, Addis Ababa.

[8] Hirvonen K. A., Bossuyt A. & Pigois R. (2017). Complementarities between social protection and health sector policies: Evidence from the productive safety net program in Ethiopia. Washington, DC and Addis Ababa, Ethiopia: International Food Policy Research Institute (IFPRI) and Ethiopian Development Research Institute (EDRI).

[9] McGaugh J. (2018) HSFR/HFG project activities and results summary: August 2013 through December 2017. Bethesda, MD: Health Finance and Governance Project, Abt Associates.

[10] Ethiopia Health Insurance Agency (2020). CBHI Members’ Registration and Contribution 2011–2020 GC. CBHI Trend Bulletin, 2020.

[11] Subrata Routh (2021). Community Based Health Insurance (CBHI) in Ethiopia: accomplishments so far and issues going forward, January 2021.

[12] Mebratie A. D., Sparrow R., Yilma Z., Alemu G., & Bedi A. S. (2015). Dropping out of Ethiopia’s community-based health insurance scheme. *Health Policy and Planning*, 30(10), 1296–1306. doi: 10.1093/heapol/czu142 25616670

[13] Mebratie A.D., Sparrow R.A., Alemu G., & Bedi A.S. (2013). Community-based health insurance schemes: A systematic review. *ISS Working Paper Series/General Series*, 568, 1–47.

[14] Mebratie, A.D. (2015). Essays on evaluating a community-based health insurance scheme in rural Ethiopia. ISS PhD Theses. International Institute of Social Studies of Erasmus University (ISS).

[15] Giedion U., Alfonso E., & Diaz Y. (2013). The impact of universal coverage schemes in the developing world: A review of the existing evidence. *Universal Health Coverage (UNICO) studies series*, *25*. Washington, DC: World Bank Group.

[16] Lagomarsino G., Garabrant A., Adyas A., Muga R., & Otoo N. (2012). Moving towards universal health coverage: Health insurance reforms in nine developing countries in Africa and Asia. *The Lancet*, 380(9845), 933–943. doi: 10.1016/S0140-6736(12)61147-7 22959390

[17] Spaan E., Mathijssen J., Tromp N., McBain F., ten Have A., & Baltussen R. (2012). The impact of health insurance in Africa and Asia: a systematic review. *Bulletin of the World Health Organization*, 90(9), 685–692. doi: 10.2471/BLT.12.102301 22984313 PMC3442382

[18] Lu C., Chin B., Lewandowski J.L., Basinga P., Hirschhorn L.R., Hill K., et al. (2012). Towards universal health coverage: An evaluation of Rwanda Mutuelles in its first eight years. *PLoS ONE* 7(6), e39282. doi: 10.1371/journal.pone.0039282 22723985 PMC3377670

[19] Schneider P. (2005). Trust in micro-health insurance: An exploratory study in Rwanda. *Social Science & Medicine*; 61(7), 1430–1438. doi: 10.1016/j.socscimed.2004.11.074 16005778

[20] Turcotte-Tremblay A. M., Haddad S., Yacoubou I., & Fournier P. (2012). Mapping of initiatives to increase membership in mutual health organizations in Benin. *International Journal for Equity in Health*, 11(1), 74. doi: 10.1186/1475-9276-11-74 23217438 PMC3541096

[21] Dror D. M., Chakraborty A., Majumdar A., Panda P., & Koren R. (2016). Impact of community-based health insurance in rural India on self-medication & financial protection of the insured. *The Indian journal of medical research*, 143(6), 809–820. doi: 10.4103/0971-5916.192075 27748307 PMC5094122

[22] Criel B., & Waelkens M. P. (2003). Declining subscriptions to the Maliando Mutual Health Organisation in Guinea-Conakry (West Africa): What is going wrong? *Social Science & Medicine*, 57(7), 1205–1219. doi: 10.1016/s0277-9536(02)00495-1 12899905

[23] Panda P., Dror I., Koehlmoos T., Hossain S., John D., Khan J., et al. (2016). Factors affecting uptake of voluntary and community-based health insurance schemes in low-and middle-income countries: A systematic review. 3ie Systematic Review 27. London: International Initiative for Impact Evaluation (3ie).

[24] Basaza R., Criel B., & Van der Stuyft P. (2008). Community health insurance in Uganda: Why does enrollment remain low? A view from beneath. *Health Policy*, 87(2), 172–184. doi: 10.1016/j.healthpol.2007.12.008 18280608

[25] Ethiopia Ministry of Health. (2016). Ethiopian national health care quality strategy: Transforming the quality of health care in Ethiopia 2016–2020.

[26] Woldemichael A., Gurara D., & Shimeles A. (2019). The impact of community-based health insurance schemes on out-of-pocket healthcare spending: Evidence from Rwanda, *IMF Working Paper*, 19(38).

[27] Donabedian A. (2005). Evaluating the quality of medical care. *The Milbank Quarterly*, 83(4), 691–729. doi: 10.1111/j.1468-0009.2005.00397.x 16279964 PMC2690293

[28] Ethiopian Health Insurance Agency. (2015). Evaluation of community-based health insurance pilot schemes in Ethiopia: Final Report.

[29] Health Statistics and Information Systems, WHO. (2015). Service availability and readiness assessment, reference manual and implementation guide.

[30] Public Health Institute [Ethiopia]. Ethiopia Service Provision Assessment Plus Survey 2014. Addis Ababa; Ethiopia, 2014.

[31] De Allegri M., Kouyaté B., Becher H., Gbangou A., Pokhrel S., Sanon M., et al. (2006). Understanding enrolment in community health insurance in sub-Saharan Africa: a population-based case-control study in rural Burkina Faso. *Bulletin of the World Health Organization*, 84(11), 852–858. doi: 10.2471/blt.06.031336 17143458 PMC2627536

