# Supplementary material for: The interaction of healthcare service quality and community-based health insurance in Ethiopia
Source: PLoS One. 2021 Aug 19;16(8):e0256132. doi: 10.1371/journal.pone.0256132 (PMC8376052; doi:10.1371/journal.pone.0256132)
Supplement: S1 File — (PDF) [file pone.0256132.s002.pdf]

Facility survey individual domain analysis

Basic Amenities:

For the basic amenity's domain, health centers were assessed based on the availability of the following tracer items: power (grid or generator), communication equipment, consultation room, improved water source within 500m of facility, adequate sanitation facilities, and emergency transportation. The study excluded having a computer with e-mail/internet access due to lack of availability in most health centers.

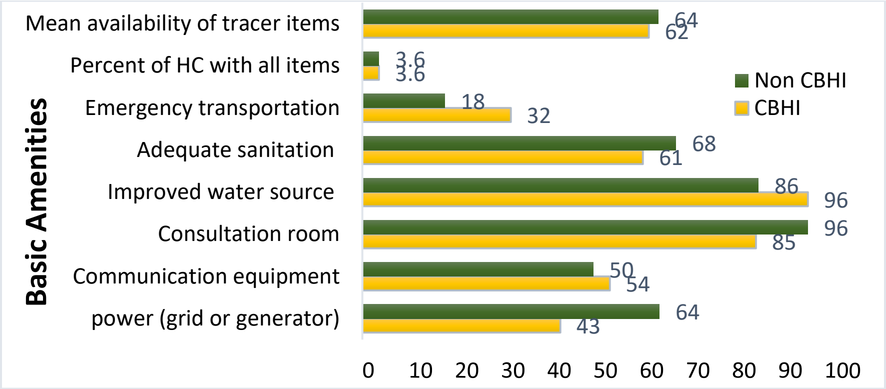

Figure 1: Percentage of Health Centers Located under CBHI and Non CBHI Woredas with Basic Amenity Items Available (N=56)

Basic Equipment:

For the basic equipment domain, the health centers were assessed on the availability of the following six tracer items: adult scale, infant scale, stethoscope, thermometer, blood pressure apparatus, and a light source for patient examinations.

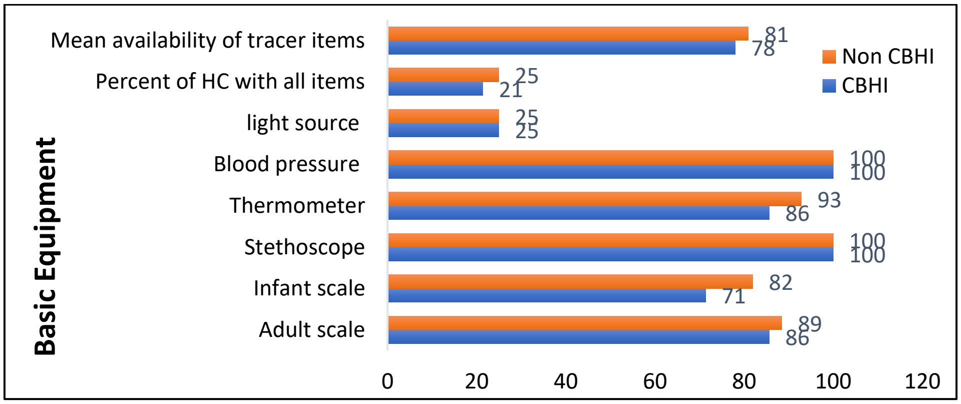

Figure 2: Percentage of Health Centers Located Under CBHI and Non CBHI Woredas with Basic Equipment Items Available (N=56)

Diagnostic Test Capacity:

For the diagnostic test capacity domain, health centers were assessed on the capacity to conduct the following seven diagnostic tests on-site: hemoglobin, blood glucose, malaria diagnostic capacity, urine dipstick with microscope (protein and Glucose), HIV diagnostic capacity, syphilis RDT (RPR), and urine pregnancy test.

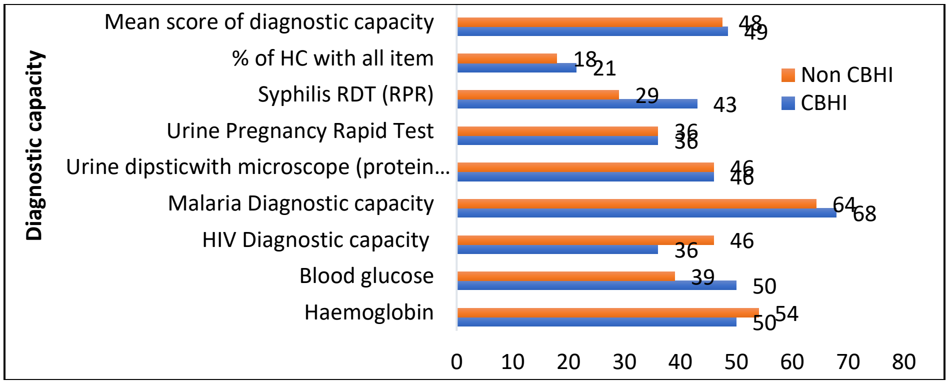

Figure 3 Percentage of Health Centers Located under CBHI and Non-CBHI Woredas with Basic Diagnostic Capacity Items Available (N=56)

Essential Medicines/Tracer Drugs:

The essential medicines list, in this context, consists of the following 25 tracers drugs expected in the health center per the national standard.

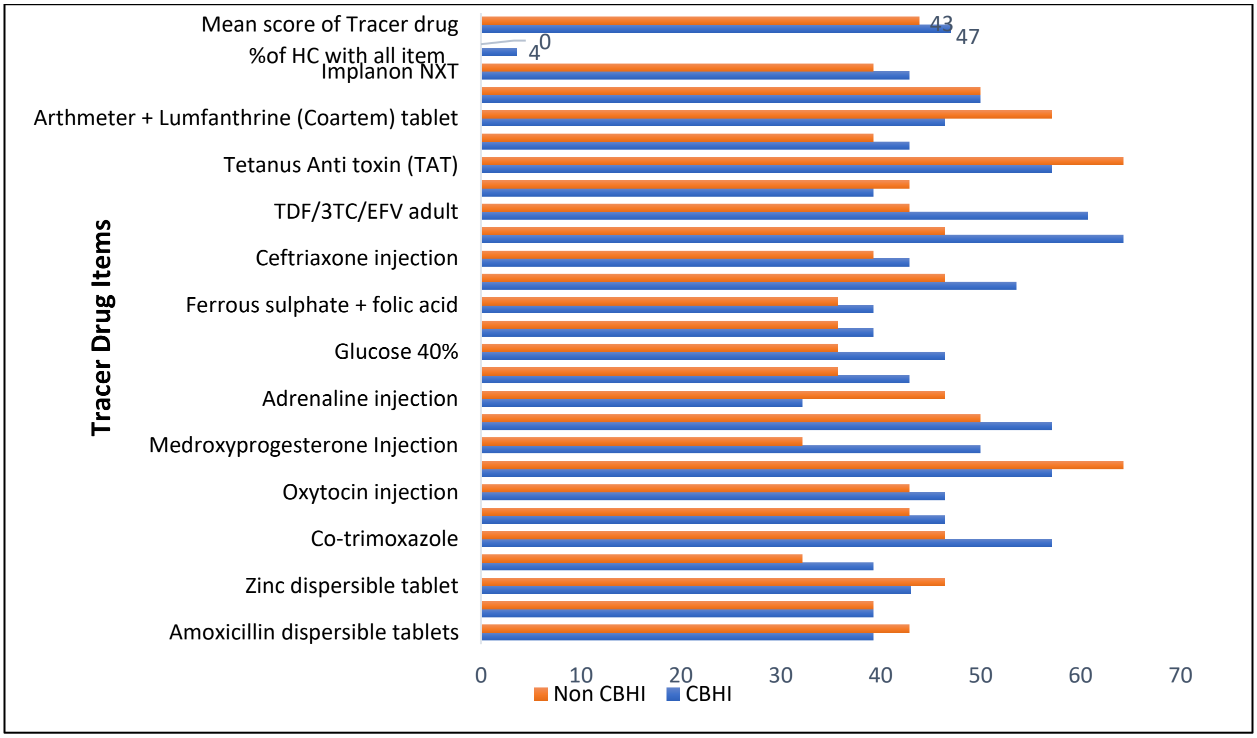

Figure 4 Percentage of Health Centers Located in CBHI and Non-CBHI Woredas with Selected Essential Medicines Items Available (N=56)

Standard Precautions for Infection Prevention:

The following nine tracer items were included in the standard precautions for infection prevention domain: sterilization equipment, disposal of sharps and other infectious wastes, disinfectant, sharps box/container, single use-standard disposable or auto-disable syringes, soap or hand disinfectant, latex gloves, masks, and guidelines for standard precautions.

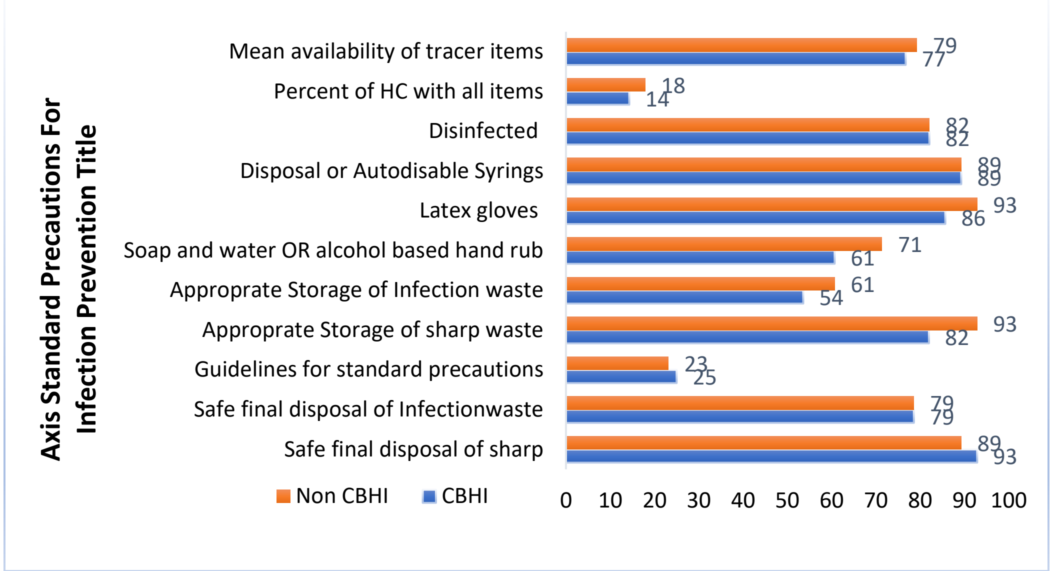

Figure 5: Percentage of Health Centers Located in CBHI and Non-CBHI Woredas with Standard Precautions for Infection Prevention (N=56)

General Service Readiness Summary Score:

The general health service readiness score is a composite summary measure designed through combining information from the five general service readiness domains. For each domain, the average availability of tracer items is the domain score.

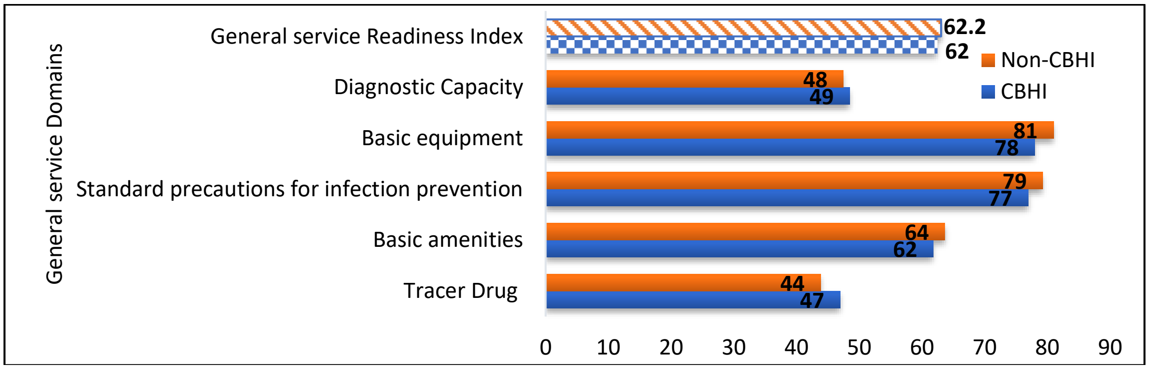

Figure 6. General Service Readiness Index and Domain Scores (N=56)

Process Measures of Quality

Followed Service Quality Standards Index

Followed service quality standard index was composed of six types of quality standard measure. Here are variations across the woredas identified for quality monitoring and patient safety practice. To illustrate its variations, Figure 3 shows the distribution of the six quality standard measures by CBHI and non-CBHI woredas. On average, 54% of the health centers followed the service quality standards index measures.

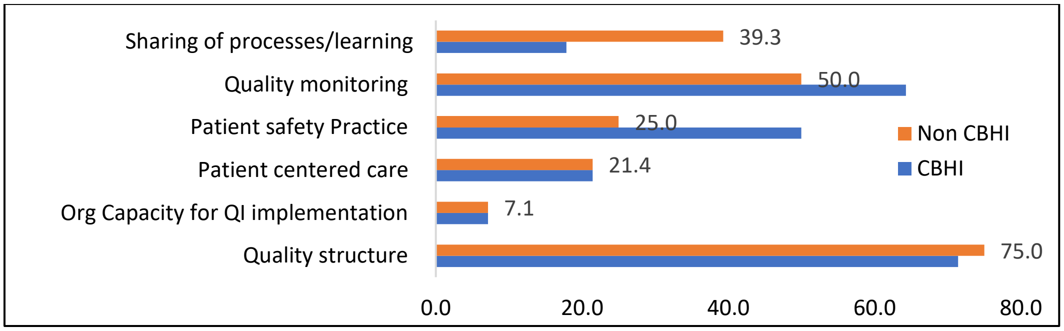

Figure 7. Average Percentage of Health Centers Located in CBHI and Non-CBHI Woredas with Standards Used for Health Care Quality (n= 56)

Perception on Interpersonal Relations

How did patients perceive the health care providers regarding compassion, respectfulness, welcoming, and responsiveness?

Table 1. Patients' Level of Perception about Interpersonal Communication with Service Provider (%)

Patient Perception About Respectful Care	CBHI			Non CBHI		
	Agree	Neither Agree nor Disagree	Disagree	Agree	Neither Agree nor Disagree	Disagree
Responsive	92.3	5.3	2.4	90.6	5	4.3
Welcoming	94.9	3.2	1.9	91.4	6.4	2.1
Respectful	94.7	3.9	1.4	91.5	5.7	2.8
Compassionate	94.7	4.4	1.0	92.9	5	2.1
Overall perception of the clients about respectful care		P=0.077				

Client' Perception About Proper Procedures Followed by Service Providers

Table 2. Practice of Procedures at Health Centers (Proportions)

Practice of Procedures	CBHI N = 415	Non-CBHI N= 141
Weight Measured	19.3	23
Temperature Taken	25.4	29.7
Use of Stethoscope	29.1	30.9
Examination (Stomach, Ear Throat etc.)	47.6	57.4
Asked about History of Illnesses	79.7	73
Asked about Symptoms	89.4	88.3
Asked about Other Treatment	49.5	55.5
Explain Diagnosis	56.5	46.7
Allowed you to ask Questions	46.9	50.7
Informed regarding your illness	57.7	52.6
Over all procedure followed	P=0.888	
