# Supplementary material for: The interaction of healthcare service quality and community-based health insurance in Ethiopia
Source: PLoS One. 2021 Aug 19;16(8):e0256132. doi: 10.1371/journal.pone.0256132 (PMC8376052; doi:10.1371/journal.pone.0256132)
Supplement: S2 File — (PDF) [file pone.0256132.s003.pdf]

KEY INFORMANT INTERVIEW

I. Regional, Zonal and Woreda Health Office Heads

INSTRUCTION TO THE INTERVIEWER

This document is meant to be used as a general guide for the CBHI and healthcare service quality intervention team during interviews with a broad range of stakeholders. Find the manager or the most senior person responsible at this level. After introducing yourself and greetings, explain briefly the purpose of the interview, the interview process and thank them for seeing us.

Provide the following information and obtain verbal informed consent to take part in the interview. The purpose of this assessment is to gather data to assess the mutual effect between CBHI and healthcare service quality in different perspectives including improving financial access, quality of health services, and community perception. This assessment will also provide recommendations for the integration of CBHI and healthcare service quality intervention at national level. If there are questions for which someone else is the most appropriate person to provide that Information, I would appreciate if you introduce me to that person. Any information you will provide as part of this interview will be held strictly confidential. Any reference to the information you provide in our analysis will be made without mentioning or implicating your name in any way.

Interviewee (Name and Title):________________________________
Region:___________________________
Zone:___________________________
Woreda:_________________________
Date:__________________________
Tel No.:___________________________

Roles and Responsibilities

1. What are the roles and responsibilities of various levels of government authorities with regards to CBHI policy making, design and management and also service quality intervention?
    a. Regional government/BOFED/RHB
    b. Regional steering committee
    c. Zonal authorities
    d. Woreda government/WOFED/WorHO

Have all been fully engaged in the process? If not, what can be done to better engage these authorities during the future scale up?

2. How do you assess the policy guidance, financial and technical support received from FMOH and EHIA? How about commitment and support from regional government in terms of budget allocation for general and targeted subsidy?
3. Who else is providing the required technical support?

Experience and Impact of the Schemes

4. Given the experiences of the CBHI woredas, what were the strength and weaknesses regarding the

a. Legal frameworks,
b. Directives, by-laws, manuals and guidelines,
c. Sensitizing the community
d. The structures and recruitment of staff
e. Providing quality service

4. The CBHI scheme design was implemented and tested for some time now. What are the major successes and challenges in the scheme parameters:
    a. Benefit package?
    b. Level of premiums?
    c. Membership scenario (HH vs. individual basis) Reimbursement amounts?
    d. Referral mechanism?
    e. Payment to the health facilities on timely manner?
    f. Enabling the schemes to cover all health related expenses through its income?
    g. Affordability to member?
    h. Fairness compared to benefit package?
    i. CBHI management schemes?
    j. Institutional arrangement (staff size, dual assignment, etc.,)?
    k. Staff skills and capacity?
    l. Quality of service

What are the major complaints of members in this regard, if any?

6. What are the successes and challenges in mobilizing the community to enroll/renew membership in CBHI? What proportion of the woreda population is currently enrolled? What strategy has worked and what hasn't?
7. Has the targeted (and general) subsidy allowed CBHI woredas to adequately include indigents in the CBHI? Are there indigents who are left out? And how significant are they in number? To what extent do you see CBHI as one of the mechanisms to increase access to health care quality? Discuss challenges in this regard, if any.
8. Could you tell us about the successes, challenges and the areas that need improvement regarding the defined benefit package and the views of CBHI members on its coverage and adequacy as well as availability of these services quality in the health facilities?
9. How successful has the CBHI scheme been in negotiating agreeable terms and contract with service providers – in terms of service quality, fee, and reduction in unnecessary services/prescription (moral hazard) etc.? What are the successes and challenges in contract administration?
10. Does the implementation of the CBHI scheme have any impact (positive or negative) on the health facilities in terms of increasing resources, improving quality of care, motivation of the staff?

<u>Health Service Utilization and Quality</u>

11. Have you seen any difference between CBHI woredas and non CBHI Woredas in terms of utilization of services, and quality of healthcare service? If there is increase in patient flow, how successful have facilities been in coping with this demand surge? How about coping with further demand increase with the scale up? Please provide evidence.
12. How is the referral of the CBHI members being carried out? Any specific challenges given that they are likely to claim preferential treatment? Any specific measures introduced? Lessons learned for scaling up.

13. To what extent are health facilities providing quality health care services for CBHI schemes as well as other clients? How does your organization support facilities to make them respond to increased demand for quality care?

Management

14. The management of the schemes is heavily dependent on the scheme managers. How do you view CBHI management, CBHI and quality of care management and staffing structure? Any successes and challenges regarding retention and motivation of CBHI, quality management team? What should non CBHI woreda learn in this regard?
15. What is the role of the woreda administration in enrolment drive, allocation of resources and staff recruitment? What worked and what didn't? What are the innovative strategies in successful woredas that should be lesson?

Financial Status

16. If the surplus of the CBHIs is increasing over time, why is this so? Are the beneficiaries not utilizing services? Is the user fees paid too low? Or are the premiums higher? Can you explain this for us?
17. Have the regional health bureau/Woreda health office invested any additional resources on health facilities (human resources, water and electricity, other equipment) to ensure that CBHI members get quality services? If yes, please describe the investments made
18. What was the role of the FMOH in improving quality of care and CBHI scheme in the CBHI woredas? Please describe the support you received from FMOH for the CBHI schemes?

2. Health Centers

INSTRUCTION TO THE INTERVIEWER

This document is meant to be used as a general guide for the CBHI and healthcare service quality intervention team during interviews with a broad range of stakeholders. Find the manager or the most senior person responsible at this level. After introducing yourself and greetings, explain briefly the purpose of the interview, the interview process and thank them for seeing us.

Provide the following information and obtain verbal informed consent to take part in the interview. The purpose of this assessment is to gather data to assess the mutual effect between CBHI and healthcare service quality in different perspectives including improving financial access, quality of health services, and community perception. This assessment will also provide recommendations for the integration of CBHI and healthcare service quality intervention at national level. If there are questions for which someone else is the most appropriate person to provide that Information, I would appreciate if you introduce me to that person. Any information you will provide as part of this interview will be held strictly confidential. Any reference to the information you provide in our analysis will be made without mentioning or implicating your name in any way.

Interviewee (Name and Title):________________________________
Region:___________________________
Zone:___________________________

Woreda:_________________________<br><br>**Name of health facility:____________________**<br><br>Date:___________________________<br><br>Tel No.:____________________________

Tell us a little about your health center (beds, services, area of service, population covered, and number of staff, size and makeup of the facility governance body)?

1. Are you a provider of health services to CBHI scheme members in the woreda? If yes, did you sign health service provision contract agreement with CBHI scheme?
2. When did you start providing health care services to CBHI beneficiaries?
3. How ready was your facility to provide services for CBHI beneficiaries?
4. What was the impact of CBHI on your facility? Did you observe significant change on demand for health care in your health facility due to CBHI?
5. Were you overwhelmed by increase demand and health seeking behavior?
6. In your opinion, what is the impact of the CBHI on utilization and quality of services? How about impact on quality of services? Give evidence
7. How do you perceive CBHI in terms of creating additional demand for health care – do you perceive it as creating additional workload and pressure to the health facility or as creating an opportunity to strengthen the capacity of your facility?
8. What incentives and disincentives CBHI schemes created on the facility and your staff?
9. After the establishment of CBHIs, are there differences in members and non-members in claiming their rights-i.e. requesting for better service? Please elaborate.
10. The CBHIs have their benefit packages. Are you able to provide all the services listed in the benefit package (for your level) to members of the CBHIs? If not what are the major gaps?
11. What are the major complaints from CBHI members on the quality of your services?
12. What is payment modality you are using to get reimbursements from CBHI members (fee for service or capitation)? If there is a difference between the payment modes of CBHI schemes and Non-members? Which mode of payment is advantageous for the health facility and why?
13. How frequently do you request and collect reimbursement for the expenses you incurred for CBHI members? Do you face any challenge in the process?
14. What is the impact of CBHIs in increasing your retained fees?
15. What kind of support did you receive from RHB and FMOH to improve quality of care because you are a CBHI provider?
16. What do you think RHB and FMOH should do for health facilities in the CBHI woredas to help them meet quality of care requirement? How about to cope with the surge in health service demand?

CBHI Management Team

INSTRUCTION TO THE INTERVIEWER

This document is meant to be used as a general guide for the CBHI and healthcare service quality intervention team during interviews with a broad range of stakeholders. Find the manager or the most senior person responsible at this level. After introducing yourself and greetings, explain briefly the purpose of the interview, the interview process and thank them for seeing us.

Provide the following information and obtain verbal informed consent to take part in the interview. The purpose of this assessment is to gather data to assess the mutual effect between CBHI and healthcare service quality in different perspectives including improving financial access, quality of health services, and community perception. This assessment will also provide recommendations for the integration of CBHI and healthcare service quality intervention at national level. If there are questions for which someone else is the most appropriate person to provide that Information, I would appreciate if you introduce me to that person. Any information you will provide as part of this interview will be held strictly confidential. Any reference to the information you provide in our analysis will be made without mentioning or implicating your name in any way.

Interviewee (Name and Title):_________________________

Region:____

Zone:____

Woreda:_______________________________________

Date:____

Telephone No:__________________________________

Tell us a little about your functions in the management of the CBHI scheme?

Enrollment

1. What is the status and progress of the woreda in enrolling its eligible residents into CBHI? What are the successes and challenges?
2. What do you think are the major reasons for some people not to enroll into the scheme or failure to renew membership?

Service Utilization and Reimbursement

3. How far CBHI members are using the health service in the contracted facilities? Do you think most of the members are using the services in the recommended (referral system) manner?
4. How often do you reimburse health facilities for services used by CBHI members? What are the mechanisms by which you check whether the invoices sent from the health facilities are right? Do you have adequate capacity to check on health facilities? Are there instances by which health facilities tried to overstate the reimbursement request amount?
5. Do you face a problem of unnecessary care seeking behaviour by CBHI members and unnecessary or over prescription of services including drugs, diagnostics etc. by health care providers (client and provider moral hazard)? Discuss how such circumstances, if they exist, affect the financial viability of the scheme?

Service Quality and Patient Satisfaction

6. Are there mechanisms whereby you are able to check on the patient perceived quality of service in contracted health facilities i.e. waiting time, availability of staff, availability of services, drugs and supplies etc.? Discuss. If so, are these regular checks or in response to

complaints from your members? Do your findings show any change in service quality? Cite examples

7. Is there any other organization that conducts quality check up on health facilities? Do you get feedback from such an organization?
8. How do you assess members' satisfaction about the services provided by the schemes and also health service providers? Do you have a standard client compliant management mechanism? Describe how, if at all, action is taken based on feedbacks? What are the major complaints forwarded by your members?

Organizational Status

9. How successful has the CBHI scheme been in recruiting and retaining core staff? Discuss successes and challenges in this regard.
10. How frequently does the Board of Management meets? What are the average attendance ratios of Board members?
11. What is the support you are receiving from RHB, WorHO, woreda administration and USAID Transform: primary health care project regional office (training, supervision, administrative support etc.)? Are you satisfied? What needs to improve?
12. What is your overall assessment of the schemes? What do you recommend for the future in terms of organizational structure, staffing, and budgeting, key design issues etc?
