# Supplementary material for: The interaction of healthcare service quality and community-based health insurance in Ethiopia
Source: PLoS One. 2021 Aug 19;16(8):e0256132. doi: 10.1371/journal.pone.0256132 (PMC8376052; doi:10.1371/journal.pone.0256132)
Supplement: S3 File — (PDF) [file pone.0256132.s004.pdf]

EXIT INTERVIEW QUESTIONNAIRE

Cover Section A: Informed Consent

Instruction to Interviewer:

Meet with the Facility Head or any other person in charge and explain the purpose of the study and inform them that you want to conduct patient exit interview at the outpatient level (at health center) and request an agreement to conduct one. Identify 5 CBHI members and 5 non-members (outpatient) and conduct interview.

Once you meet with the interviewee use the consent for below:

የቅድመ መረጃ ፍቃድ

ጤና ይስጥልኝ! በቅድሚያ ለምንጠይቅዎት ጥያቄዎች መልስ ለመስጠት ጊዜዎን በመስጠትዎ እናመሰግናለን፡፡ መንግሥት ባለፉት ዓመታት በወረዳው ውስጥ የማኅበረሰብ አቀፍ የጤና መድህን መጀመሩ ይታወቃል፡፡ ይህ ፕሮግራም በኅብረተሰቡ የጤና አጠቃቀም፤ የአገልግሎት ጥራትና ወጭን በመቀነስ ረገድ ያሳደረውን ለውጥ በማጥናት ላይ እንገኛለን፡፡ የማኅበረሰብ አቀፍ የጤና መድን እንዴት የአገልግሎት ጥራትን መሻሻል እንዳለበት የእርስዎን አስተያየት እንጠይቃለን፡፡ እርሰዎ በአጋጣሚ ተመርጠዋል፡፡ መጠይቁ 20 ደቂቃ ይፈጃል፡፡ የሚሰጡት ምላሽ ሁሉ ሚስጢራዊ ሲሆን ከአማካሪዎቹ እና ከቃለ መጠይቅ አድራጊው ውጪ ጥቅም ላይ አይውልም፡፡ በዚህ ጥናት የሚሳተፉት <u>በፈቃደኝነት</u> ብቻ ነው፡፡ በዚህ ጥናት በፈቃደኝነት ለመሳተፍ መስማማትዎን በቃል እንዲያረጋግጡልኝ በአክብሮት እጠይቃለሁ፡፡

Cover Section B: Background

1. Name Of the region ________________
2. Name of the Woredas ________________________
3. Name of the Health centers ________________________
4. Name of Interviewer: ________________________________
5. Date: | | | Day | | | Month | | | | | Year

6. Patient No. ______________________________________

	Section1: Patient's Background (Both CBHI/Non-CBHI woreda Respondents)	
Sr.No	Questions	Response
1.1	Age (in completed years)	__ | **years**
1.2	Sex (fill as observed)	1=Male<br>2=Female
1.3	Highest educational attainment	1=Illiterate<br>2=reading and writing<br>3=primary education (Grad1-6)<br>4=secondary education (Grade7-12)<br>5=vocational training<br>6=tertiary education<br>7=under age
1.4	Residence	1=urban<br>2=rural
1.5	Marital status	1=never married<br>2=married<br>3=living together<br>4=divorced/separated<br>5=widowed
1.6	Occupation/employment	1=employed/self-employed(E.g. farming)<br>2=non-employed (above 18 years<br>3=student<br>4=other, specify___________
1.7	What is your ethnicity?<br>Record their ETHNIC GROUP	ETHNICITY ---------------------
1.8	How many members of family do you share your meals with? HH family size	(number)
1.9	Estimated annual income in birr (ETB)	(estimated in birr)
1.10	Within the last year (12 months) how much money did you spend getting health care?	(number)
1.11	Have you had to borrow money for getting health care (for you and your family in the HH) in the last 12 months?	(estimated in birr)
	**Section2: Service utilization and quality(Both CBHI/Non-CBHI woreda Respondents)**	
2.1	For how long have you used this health facility? (**state number of months or years**)	**|___|months**<br>**|___|years**
2.2	What were the reasons for you visit today? **(multiple response is possible)**	1=Diarrhea<br>2=Fever<br>3=Respiratory problem<br>4=Cough<br>5=Nausea/vomiting<br>6=Head ache<br>7=Stomachache<br>8=Toothache<br>9=Joint/muscle pain<br>10=Delivery<br>11=Injury<br>12=eye problem<br>13=other(specify)___________
2.3	How long did it take you to get here from your home?	(in minutes/hours)
2.4	What transport did you use to get here	1=Ambulance<br>2=Public transport<br>3=On foot<br>4=Other specify ……….

2.5	Which services did you obtain during this visit/stay? **(multiple response is possible)**	1=Consultation/card<br>2= Diagnosis (lab and others)<br>3=Drugs and medical supplies<br>4= Inpatient services (bed/food)<br>5=Delivery<br>6=Surgical procedure<br>7=others, specify
2.6	If the answer to question 2.5 is 2 and/or 3, were you able to get the Prescribed drugs/supplies?	1= fully (all prescribed)<br>2= partially (only some of them)<br>3= none (go to question 2.8)
2.7	If the answer to question 2.6 is 1 or 2, where did you get it from? **(multiple response is possible)**	1= within the visited facility<br>2= being sent to other CBHI contracted facilities (including drug retailers)<br>3= being sent to other facility/drug stores/diagnostic center
2.8	If the answer to question 2.6 is 2 and or 3, what is the reason?	1= unavailability within the facility visited<br>2= unable to buy

Sr.No	Questions	Response
2.9	How much money you have spent to come here and get back to your home?	Transportation ……………<br>Lodging /food ……………<br>Medical/ Surgical Service ………<br>Drug ………………………<br>Others (specify) …………<br>Total Cost ………………………
2.10	How satisfied are you with the service you received during this visit/stay? (Indicate the code for questions 2.10.1-2.10.7)	1=Very satisfied<br>2=satisfied<br>3=Neither satisfied nor dissatisfied<br>4=dissatisfied<br>5=Very dissatisfied
2.10.1	Overall quality of service?	|____________|
2.10.2	Availability of drugs/medical supplies?	|____________|
2.10.3	Availability of diagnostic facilities?	|____________|
2.10.4	Cleanliness of the facility?	|____________|
2.10.5	Waiting time (from the time of arrival in the health facility up to seeing a health professional?	|____________|
2.10.6	Waiting time between services (e.g. between consultation and until completing the treatment/diagnosis)?	|____________|
2.10.7	Friendliness of staff?	|____________|
2.10.8	How did you feeling of staff respect	|____________|
2.11	Have you observed the quality of care, consultation, and diagnostics care service you received? (Indicate the code for questions 2.11.1-2.11.8)	1=Yes,<br>2=No
2.11.1	Does your health care provider measure your weight?	|____________|
2.11.2	Does your health care provider measure your body temperature?	|____________|

2.11.3	Does your health care provider use a stethoscope?	|____|
2.11.4	Does your health care provider make proper examination(touch the stomach, ear, throat, etc)	|____|
2.11.5	Does the health care provider asked you about the history of the illness?	|____|
2.11.6	Does the health care provider asked you about your symptoms?	|____|
2.11.7	Does the health care provider asked if treatment was taken before arrival at the facility	|____|
2.11.8	Does the health care provider explained to you the diagnosis	|____|
2.12	Perceived quality of care by Domain (Indicate the code for questions 2.12.1-2.12.17)	1=Strongly Agree<br>2=Agree<br>3=Neither agree nor disagree<br>4=disagree<br>5=Strongly Disagree
**Perceived availability of health care providers, supplies, and physical resources**		
2.12.1	Medical supplies and equipment are sufficient	|____|
2.12.2	Rooms are sufficient	|____|
2.12.3	Adequate/appropriate health care providers for women	|____|
2.12.4	There is sufficient high quality health care providers	|____|
2.12.5	Availability of laboratory service	|____|
2.12.6	Medicine for all illnesses is always available	|____|
	**Perceived quality of health care delivery.**	
2.12.7	Health care providers conduct quality diagnostic exams	|____|
2.12.8	Health care providers make appropriate drug prescriptions	|____|
2.12.9	The quality of drugs prescribed is good	|____|
2.12.10	Treatment provided is efficient and effective	|____|
	**Perceived quality of health care provider conduct**	
2.12.11	Health care providers show compassion and support for patients	|____|
2.12.12	Health care providers are respectful to patients	|____|
2.12.13	Health care providers provide quality follow-up care	|____|
2.12.14	Health care providers are welcoming during consultations	|____|
2.12.15	Health care providers respect patient confidentiality	|____|
2.12.16	Facility assistants are friendly and helpful to patients	|____|
2.12.17	Facility assistants respond to patients questions	|____|
2.13	For how long did you wait before you had consultation with staff today?	1= Less than 30 minutes<br>2=30 to 60 minutes<br>3= 1 to 3 hours<br>4=3 to 6 hours<br>5= 6 hours and more
2.14	For how long did you wait between services (e.g. between consultation and diagnosis) today to utilize various services?	1= Less than 30 minutes<br>2=30 to 60 minutes<br>3= 1 to 3 hours<br>4=3 to 6 hours<br>5= 6 hours and more
2.15	Is this health facility your preferred service point for your future health care needs?	1=Yes because I am satisfied with the service quality<br>2= Yes because I do not have another option

		3= No		
2.16	Are you satisfied about the quality of healthcare service provided by the service providers	1=Yes<br>2=No		
2.17	How much did you pay for the service you obtained (in ETB)? (for non-members and members who utilized a service which is not covered by CBHI (ask the patient the type of service they acquired check against the list of CBHI service package)	1=Consultation------------<br>2=Diagnosis-----------------<br>3=Drugs and medical supplies--------------<br>4= Inpatient services------------------<br>5= Transport *(for inpatient only)*---------<br>*6=Laboratory test*-------------<br>6=others, specify--------------		
2.18	Have you ever heard about community based health insurance program (CBHI)? ***[non-CBHI members only]***	1=Yes<br>2=No (skip 2.19)		
2.19	From whom did you hear about CBHI?	1= neighbors/friends<br>2= CBHI officials in public meeting<br>3= CBHI house to house awareness creation campaigns<br>4= mass media: ETV, radio<br>5= health professionals in health facilities<br>6=others, specify________________________		
3.20	Perceptions about CBHI [**put ticks on the space provided]**	Correct (1)	Not correct (2)	Do not know (3)
3.20.1	Only those who fall sick should consider enrollment in CBHI			
3.20.2	Only the very poor who cannot afford to pay for healthcare need to join the schemes			
3.20.3	Under CBHI program, you pay money (premiums) in order for the CBHI to finance your future health care needs?			
3.20.4	CBHI program are like savings scheme, you will receive interest and get your money back			
3.20.5	If you do not make claims through CBHI, your premium will be returned			

Section3: CBHI Related (Only CBHI Woreda respondents)		
Sr.No	Questions	Response
3.1	Are you ever enrolled in a CBHI scheme in your woreda? (HH /person enrollment)	1=Yes<br>2=No (go to 3.14)
3.2	When did you become a CBHI member?	Write the year |____|
3.3	Why did you decide to enroll in CBHI? (multiple response is possible)	1=Illness and/or injury occurs frequently in our household<br>2= To finance health care expenses<br>3= household is exempt from registration fee and premium payment<br>4=Premium is low compared to the user fee price to obtain medical treatment<br>5= Pressure from other family members/community<br>6= Pressure from the CBHI office<br>7= Others, please specify
3.4	How do you feel about the adequacy of the benefit package?	1=very adequate<br>2= somewhat adequate<br>3= inadequate<br>4=I do not know
3.5	Have you observed any improvement in service quality since this health facility has been contracted by the CBHI scheme(mention the year (to be asked CBHI woredas only) put code as appropriate for questions 3.5.1 to 3.5.7)	1=Yes<br>2=No<br>3=Don't observe
3.5.1	Overall quality of service?	|____________|
3.5.2	Availability of drugs/medical supplies?	|____________|
3.5.3	Availability of diagnostic facilities?	|____________|
3.5.4	Cleanliness of the facility?	|____________|
3.5.5	Waiting time (from the time of arrival in the health facility up to seeing a health professional?	|____________|
3.5.6	Waiting time between services (e.g. between consultation and diagnosis)	|____________|
3.5.7	Friendliness of staff?	|____________|
3.6	How many times have you /your family been treated since you join the CBHI scheme?	--------------------
3.7	Are you currently active CBHI member?	1= Yes<br>2=No (go to 3.16)
3.8	When you current membership expiries, Do you have a plan to renew your CBHI membership?	1= Yes<br>2=No (go to 3.14)
3.9	The timing/time interval of premium payment is convenient to you?	1=Yes<br>2=No,
3.10	The CBHI registration fee is	1=easily affordable<br>2=somewhat affordable<br>3=unaffordable
3.11	The CBHI regular contribution (premium) is	1=easily affordable<br>2=somewhat affordable<br>3=unaffordable
3.12	CBHI payment modality	1=integrated with taxation of land<br>2=independent
3.13	If you are a CBHI member, how is service modality	1=separate room for CBHI member<br>2=integrated with other non-members
3.14	If you have decided not to renew your CBHI membership, state the reason.<br>**(multiple response is possible)**	1=Illness and injury does not occur frequently in our household<br>2=The registration fee and premiums are not affordable<br>3= There is limited availability of health services<br>4=The quality of health services is low<br>5= CBHI management staff is not trustworthy<br>6= The quality of service for CBHI members is worse than for non-CBHI members

		7= Waiting time to access services is longer for CBHI members<br>8= Other, specify
3.15	Have you benefited from the CBHI scheme?	1=Yes<br>2=No
3.16	If yes to question 3.15, how does you benefited? (**Multiple responses possible- list in order of importance)**	1= Increased access to health care<br>2 = Reduced costs of health care<br>3 = Reduced concerns about expected health care costs<br>4 = Others, please specify
3.17	If No to question 3.15, why do you think you has not benefited? (**multiple responses possible-list in order of importance)**	1 = No one in my HH has visited health facilities<br>2= We still pay other additional costs for treatment (specify)<br>3= The quality of service (waiting time, staff attitude, medicine, diagnostics) for CBHI members is not as good as for out of pocket paying patients (non-CBHI members)<br>4= Delays in issuance and distribution of CBHI ID cards<br>5=others, specify
3.18	If you are not currently a CBHI member, why did you decide not to enroll in CBHI? **(multiple response is possible)**	1=Illness and injury does not occur frequently in our household<br>2=The registration fee and premiums are not affordable<br>3=Want to wait in order to confirm the benefits of the scheme from others<br>4= We do not know enough about the CBHI scheme<br>5= There is limited availability of health services<br>6= The quality of health care services is low<br>7= The benefit package does not meet our needs<br>8= CBHI management staff is not trustworthy<br>9= Waiting time to access services is longer for CBHI members<br>10=I am fee waiver beneficiary<br>11= Other reasons, please specify<br>12= I don't know
3.19	Do health professionals treat CBHI members and non-CBHI members equally, with impartiality?	1= Yes 2= No<br>3 = don't know
3.20	Is there any gender based preference in membership to CBHI scheme?	1= Yes 2= No<br>3 = don't know
3.21	Does CBHI membership cause any advantage or disadvantage to women and girls?	1= Yes 2= No<br>3= don't know

Facility Survey

CONSENT			
FIND THE MANAGER, THE PERSON IN-CHARGE OF THE FACILITY, OR MOST SENIOR HEALTH WORKER RESPONSIBLE FOR OUTPATIENT SERVICES WHO IS PRESENT AT THE FACILITY. READ THE FOLLOWING GREETING:<br>Good day! My name is _________________. We are here on behalf of USAID Transform: primary Health care project conducting a survey of health facilities to assist the variability of service in selected facility.<br>Now I will read a statement explaining the study.<br><br>Your facility was selected to participate in this study using purposive sampling. We will be asking you questions about various health services. Information about your facility may be used by the FMOH, Transform: Primary health care project, organizations supporting services in your facility, and researchers, for planning service improvement or for conducting further studies of health services.<br><br>Neither your name nor that of any other health worker respondents participating in this study will be included in the dataset or in any report; however, there is a small chance that any of these respondents may be identified later. Still, we are asking for your help to ensure that the information we collect is accurate. You may refuse to answer any question or choose to stop the interview at any time. However, we hope you will answer the questions, which will benefit the services you provide and the nation.<br>If there are questions for which someone else is the most appropriate person to provide the information, we would appreciate if you introduce us to that person to help us collect that information.<br>At this point, do you have any questions about the study? Do I have your agreement to proceed?<br><br>____________________________<br>**INTERVIEWER'S SIGNATURE INDICATING CONSENT OBTAINED DAY MONTH YEAR**			
01	May I begin the interview?	YES ....1<br>NO 2	➔stop
02	Is facility singed agreement to provide service for CBHI members	YES----------------------1<br>NO-----------------------2	
03	Is facility in CBHI woreda?	YES----------------------1<br>NO-----------------------2	
04	Is facility targeted by TPHC QI/QA initiative?	YES----------------------1<br>NO-----------------------2	

Number	Question	Result			Skip
**SERVICE AVAILABILITY**					
**Section1: STAFFING**					
1	I have a few questions on staffing for this facility. Please tell me how many staff with each of the following qualifications are currently assigned to, employed by, or seconded to this facility. Please count each staff member only once, on the basis of the highest technical or professional qualification. For doctors, I would also like to know, of the total number, how many are part-time in this facility.	A) Assigned/ employed/ seconded (including part time)	B) Part time	C) number of professional s required per facility	
1.1	Generalist (non-specialist) medical doctors				
1.2	Nursing professionals				
1.3	Midwifery professionals				
1.4	Pharmacy professionals				
1.5	Laboratory professionals (medical and pathology)				
1.6	Health extension workers				
1.7	Health officer				
1.8	Integrated Emergency Surgical Officer (IESO)				
1.9	Other non-physician clinicians (e.g. BSc + other specialization)				
1.10	BSc anaesthetist				
1.11	Health education and promotion professional				
1.12	Emergency Medical Technician (EMT, paramedic)				
1.13	Bio-medical engineer				
1.14	Environmental health professional				
1.15	Health informatics professional				

Section 2: INPATIENT AND OBSERVATION BEDS			
**2.1**	Excluding any delivery beds, how many overnight/inpatient beds in total does this facility have, both for adults and children?	# OF OVERNIGHT/ INPATIENT BEDS. . . . . [ ][ ][ ][ ]	
**2.2**	Of the overnight/inpatient beds in this facility, how many are dedicated maternity beds?	# OF DEDICATED MATERNITY BEDS. . . . . [ ][ ][ ]	

Section 3: AVAILABLE SERVICES			
3.1	Does this facility offer family planning services now?	YES……………..1<br>NO………………2	
3.2	Does this facility offer now antenatal care (ANC) services?	YES……………..1<br>NO………………2	
3.3	Does this facility offer services for the prevention of mother-to-child transmission of HIV (PMTCT)?	YES……………..1<br>NO………………2	
3.4	Does this facility offer delivery (including normal delivery, basic emergency obstetric care, and/or comprehensive emergency obstetric care) and/or new-born care services?	YES……………..1<br>NO………………2	
3.5	Does this facility stock any medicines for obstetric care in this service site?	YES……………..1<br>NO………………2	
**3.6**	Does this facility provide CEmOC service?	YES……………..1<br>NO………………2	2 skip to 3.10
3.7	Do you have the national guidelines for Comprehensive Emergency Obstetric Care (CEmOC) available in this facility today?	YES, observed……..1<br>YES, reported not seen………..2<br>NO…………3	
3.8	Have you or any provider(s) of delivery service received any training in Comprehensive Emergency Obstetric Care (CEmOC) in the last two years?	YES……………..1<br>NO………………2	
3.9	Does this facility have a health professional who can perform caesarean section present in the facility or on call 24 hours a day (including weekends and on public holidays)?	YES……………..1<br>NO………………2	
3.10	Does this facility offer immunization services?	YES……………..1<br>NO………………2	
3.11	Have you or any provider(s) of immunization service delivery received any training in any of the following child immunization services in the last two years?	YES……………..1<br>NO………………2	
3.12	Does this facility offer preventative and curative care services for children under 5?	YES……………..1<br>NO………………2	
3.13	Does this facility offer adolescent health services?	YES……………..1<br>NO………………2	
3.14	Does this facility offer diagnosis or treatment of STIs other than HIV?	YES……………..1<br>NO………………2	
3.15	Does this facility offer diagnosis, treatment prescription, or treatment follow-up of tuberculosis?	YES……………..1<br>NO………………2	
3.16	Does this facility manage and provide treatment follow-up for TB patients?	YES……………..1<br>NO………………2	
3.17	Does this facility now stock any medicines for TB treatment?	YES, in service site……………..1<br>YES, elsewhere (e.g bulk store/pharmacy)………………….2<br>YES, in both locations………..3<br>NO, TB meds not stocked……….4	

3.18	Does this facility offer diagnosis or treatment of malaria?	YES……………..1<br>NO……………..2	
3.19	Does this facility offer any surgical services (including minor surgery such as suturing, circumcision, wound debridement, etc.), or caesarean section?	YES……………..1<br>NO……………..2	
3.20	Does this facility offer blood transfusion services?	YES……………..1<br>NO……………..2	
3.21	Does this facility conduct any diagnostic testing including any rapid diagnostic testing?	YES……………..1<br>NO……………..2	
3.22	Does this facility conduct the following blood group serology tests onsite or offsite?	YES, onsite ------------1<br>YES, offsite----------2<br>Don't conduct the test---3	
3.23	Does this facility perform diagnostic x-rays, ultrasound, or computerized tomography?	YES……………..1<br>NO……………..2	
3.24	Does this facility stock medicines, vaccines, or contraceptive commodities?	YES……………..1<br>NO……………..2	
3.25	Check the expiry dates of the stored product. Are they stored in first-to-expire, first-out (FEFO) order (i.e. the stock that will expire first is the closest to the front)?	YES……………..1<br>NO……………..2	
3.26	Does this facility now have the needed stock of medicines for malaria treatment?	YES……………..1<br>NO……………..2	
3.27	Would you say that you are currently experiencing stock-out of some essential/terser drugs/?	YES……………..1<br>NO……………..2	
3.28	How often do you face stock-out of essential drugs?	YES……………..1<br>NO……………..2	
3.29	Have you ever faced drug stock-outs of supplies when the demand for services were high	YES……………..1<br>NO……………..2	

Number	Question	Result	Skip
	**SERVICE READINESS**		
**Section 4: INFRASTRUCTURE**			
**COMMUNICATIONS**			
4.1	Does this facility have a ***functioning land line telephone*** that is available to call outside at all times client services are offered?<br>**Clarify that if facility offers 24-hour emergency services, then this refers to 24-hour availability.**	YES.................1<br>NO..................2	
4.2	Does this facility have a ***functioning cellular telephone or a private cellular phone*** that is supported by the facility?	YES.................1<br>NO..................2	
4.3	Does this facility have a ***functioning short-wave radio*** for radio calls?	YES.................1<br>NO..................2	
4.4	Does this facility have ***a functioning computer?***	YES.................1<br>NO..................2	
4.5	Is there access to email or internet within the facility today?	YES.................1<br>NO..................2	
AMBULANCE/TRANSPORT FOR EMERGENCIES			
4.6	Does this facility have a ***functional ambulance*** or other vehicle for emergency transportation for clients that is stationed at this facility or operates from this facility?	YES.................1<br>NO..................2	
4.7	Does this facility have access to an ambulance or other vehicle for emergency transport for clients that is stationed at another facility or that operates from another facility in near proximity?	YES.................1<br>NO..................2	
4.8	Is fuel for the ambulance or other emergency vehicle available today?	YES.................1<br>NO..................2<br>Don't know...98	
POWER SUPPLY			
**4.9**	Does your facility have functional electricity from any source (e.g. electricity grid, generator, solar, or other) including for stand-alone devices (EPI cold chain)?	YES.................1<br>NO..................2	
**4.10**	What is the electricity used for in the facility?	ONLY STAND-ALONE ELECTRIC MEDICAL DEVICES/APPLIANCES (e.g. EPI cold room, refrigerator, suction apparatus, etc.) ....1<br>ELECTRIC LIGHTING (EXCLUDING FLASHLIGHTS) AND COMMUNICATIONS ....2<br>ELECTRIC LIGHTING, COMMUNICATIONS, AND 1 TO 2 ELECTRIC MEDICAL DEVICES/ APPLIANCES3<br>ALL ELECTRICAL NEEDS OF FACILITY 4	

Number	Question	Result	Skip
4.11	During the past 7 days, was electricity available at all times from the main or any backup source when the facility was open for services?	Always available (no Interruptions).......1.<br>Often available (interruptions of less than 2 hours per day)... 2<br>Sometimes available (frequent or prolonged interruptions of more than 2 hours per day).... 3	

**BASIC CLIENT AMENITIES**			
4.11	On average, how many hours per day is this facility open?	4 hours or less.......... 1<br>5 to 8 hours .... 2<br>9 to 16 hours.......... 3<br>17 to 23 hours.......... 4<br>24 hours.......... 5	
4.12	What is the ***most commonly used*** source of water for the facility ***at this time***?<br><br>**Observe that water is available from the source or in the facility on the day of the visit. E.g. check that the pipe is functioning.**	Piped into facility.......... 1<br>Piped onto facility grounds .... 2<br>Public tap/standpipe .... 3<br>Tubewell/borehole.......... 4<br>Protected dug well .... 5<br>Unprotected dug well .... 6<br>Protected spring .... 7<br>Unprotected spring.......... 8<br>Rainwater collection .... 9<br>Bottled water..........10<br>Cart w/small tank/drum..........11<br>Tanker truck ....12<br>Surface water ....13<br>Other (specify) ____________ 96<br>Don't know ____________ 98<br>No water source..........00	
4.13	Is water available from this source on facility premises?	YES, inside the facility .... 1<br>YES, within the ground of the facility .... 2<br>NO, outside the facility grounds.......... 3	
4.14	Is there a room with auditory and visual privacy available for patient consultations?	Auditory privacy only.......... 1<br>Visual privacy only.......... 2<br>Both auditory and visual privacy .... 3<br>No privacy.......... 4	
4.15	Is there a toilet (latrine) on premises that is accessible for general outpatient client use? IF YES: What type of toilet?<br>**If multiple toilets are available, consider the most modern type**	Flush toilet.......... 1<br>Ventilated improved pit latrine (vip)…………2<br>Pit latrine with slab .... 3<br>Pit latrine without slab/open pit.......... 4<br>Composting toilet .... 5<br>Bucket.......... 6<br>Hanging toilet/ hanging latrine.......... 7<br>No facilities on premises/bush/field...... 8	
**INFECTION CONTROL**			
4.16	Does this facility have any guidelines on standard precautions for infection prevention?	YES, observed.......... 1<br>YES, reported not seen.......... 2<br>NO.......... 3	

HEALTH CARE WASTE MANAGEMENT			
4.17	Now I would like to ask you a few questions about waste management practices for sharps waste, such as needles or blades.<br><br>How does this facility ***finally*** dispose of sharps waste (e.g., filled sharps boxes)?<br><br>**Probe to arrive at correct response**<br><br>**Note: if any of the responses 2-9 take place outside the facility, then the correct response to circle will be in the category of "remove offsite"**	Same as for sharps items........................... 1<br>**BURN INCINERATOR**<br>2-chamber industrial (800-1000+° c) ... 2<br>1-chamber drum/brick.............................. 3<br>**OPEN BURNING**<br>Flat ground - no protection .... 4<br>Pit or protected ground .... 5<br>**DUMP WITHOUT BURNING**<br>Flat ground - no protection .... 6<br>Covered pit or pit latrine........................... 7<br>Open-pit - no protection............................ 8<br>Protected ground or pit .... 9<br>**REMOVE OFFSITE**<br>Stored in covered container....................10<br>Stored in other protected environment<br>..11<br>Stored unprotected....................................12<br>Other (specify)________________________96<br>Never has sharp waste _______________95	
4.18	Now I would like to ask you a few questions about waste management practices for medical waste other than sharps, such as used bandages.<br><br>How does this facility ***finally*** dispose of medical waste other than sharps boxes?<br><br>**Probe to arrive at correct response**<br><br>**Note: if any of the responses 2-9 take place outside the facility, then the correct response to circle will be in the category of "remove offsite"**	Same as for sharps items........................... 1<br>**BURN INCINERATOR**<br>2-chamber industrial (800-1000+° c) ... 2<br>1-chamber drum/brick.............................. 3<br>**OPEN BURNING**<br>Flat ground - no protection .... 4<br>Pit or protected ground .... 5<br>**DUMP WITHOUT BURNING**<br>Flat ground - no protection .... 6<br>Covered pit or pit latrine........................... 7<br>Open-pit - no protection............................ 8<br>Protected ground or pit .... 9<br>**REMOVE OFFSITE**<br>Stored in covered container....................10<br>Stored in other protected environment<br>..11<br>Stored unprotected....................................12<br>Other(specify) ________________________96<br>Never has sharp waste _______________95	

SUPERVISION			
4.19	Does this facility receive any external supervision?<br>**This may be from the federal, regional, zonal, woreda levels, or a higher level health facility.**	YES……………..<br>1<br>NO……………..<br>2	
4.20	When was the last time this facility received a supervision visit from the higher level (District Health Management Team or other)?	Past 30 days…………………..1<br>Past 90 days…………………..2<br>More than 90 days ago……..3<br>Don't<br>know……………………..98	

4.21	During the supervision visit, did the supervisor assess the following?	**YES**	**NO**
01	Pharmacy (e.g. drug stock out, expiry, records, etc.)	1	2
02	Staffing (e.g. staff available and training)	1	2

03	Data (e.g. completeness, quality, and timely reporting)	1	2

BASIC EQUIPMENT

4.22	Please tell me if the following basic equipment and supplies used in the provision of client services are available and functional in this facility today. **ASK TO SEE THE ITEMS**	A) AVAILABLE			
		Observed	Reported not seen	Not available	Don't know
01	Adult weighing scale	1	2	3	8
02	Child weighing scale- 250 gram gradation	1	2	3	8
03	Infant weighing scale – 100 gram gradation	1	2	3	8
04	Measuring tape-height board/stadiometre	1	2	3	8
05	Thermometer	1	2	3	8
06	Stethoscope	1	2	3	8
07	Blood pressure apparatus (may be digital or manual sphygmomanometer with stethoscope)	1	2	3	8
08	Light source (flashlight acceptable)	1	2	3	8
09	Intravenous infusion kits	1	2	3	
10	Oxygen concentrators	1	2	3	8
11	Oxygen cylinders	1	2	3	8
12	Central oxygen supply	1	2	3	8
13	Flowmeter for oxygen therapy (with humidification)	1	2	3	8
14	Oxygen delivery apparatus (key connecting tubes and mask/nasal prongs)	1	2	3	8
4.23	At any time during the past 3 months has oxygen been unavailable for any reason?	YES .... 1<br>NO 2			

INFECTION CONTROL PRECAUTIONS

4.24	Please tell me if the following resources/supplies used for infection control are available in the general outpatient area of this facility today. **ASK TO SEE THE ITEMS**	OBSERVED	REPORTED NOT SEEN
01	Clean running water (piped, bucket with tap, or pour pitcher)	1	2
02	Hand-washing soap/liquid soap	1	2
03	Alcohol based hand rub	1	2
04	Disposable latex gloves	1	2
05	Waste receptacle (pedal bin) with lid and plastic bin liner	1	2
06	Sharps container ("safety box")	1	2
07	Environnemental désinfectant (e.g., chlorine)	1	2
08	Disposable syringes with disposable needles	1	2
09	Auto-disable syringes	1	2

We have now completed all of the questions in this module of the survey. Thank you for your participation

Number	Question	Result	Skip
**Section 5 : INTERVIEWER'S OBSERVATIONS**			
5.1	**INTERVIEWER END TIME** (use the 24 hour-clock system)	☐☐ : ☐☐	
5.2	**RESULT CODES (LAST VISIT):**	Completed.. 1<br>Respondent not available.................. 2<br>Refused... 3<br>Partially completed .... 4<br>Other(specify)__________________ 96	
**COMMENTS ABOUT THE RESPONDENT:**			
____<br>____			
**COMMENTS ON SPECIFIC QUESTIONS:**			
____<br>____			
**ANY OTHER COMMENTS:**			
____<br>____<br>____<br>____<br>____			
**SUPERVISOR'S OBSERVATIONS:**			
____<br>____<br>____<br>____<br>____			
**NAME OF SUPERVISOR:** ______________________________		DATE: ____________________	
