# Supplementary material for: The interaction of healthcare service quality and community-based health insurance in Ethiopia
Source: PLoS One. 2021 Aug 19;16(8):e0256132. doi: 10.1371/journal.pone.0256132 (PMC8376052; doi:10.1371/journal.pone.0256132)
Supplement: Peer review history

# Peer review: Decision Letter 0

**DOI:** 10.1371/journal.pone.0256132.r001

**Authors:** Hafiz T.A. Khan

7 May 2021

PONE-D-21-11751

The interaction of health service quality and community-based health insurance in Ethiopia

PLOS ONE

Dear Authors,

Thank you for submitting your manuscript to PLOS ONE. After careful consideration, we feel that it has merit but does not fully meet PLOS ONE’s publication criteria as it currently stands. Therefore, we invite you to submit a revised version of the manuscript that addresses the points raised during the review process.

Please submit your revised manuscript by 5 of July 2021. If you will need more time than this to complete your revisions, please reply to this message or contact the journal office at plosone@plos.org. When you're ready to submit your revision, log on to https://www.editorialmanager.com/pone/ and select the 'Submissions Needing Revision' folder to locate your manuscript file.

Please include the following items when submitting your revised manuscript:

- A rebuttal letter that responds to each point raised by the academic editor and reviewer(s). You should upload this letter as a separate file labeled 'Response to Reviewers'.
- A marked-up copy of your manuscript that highlights changes made to the original version. You should upload this as a separate file labeled 'Revised Manuscript with Track Changes'.
- An unmarked version of your revised paper without tracked changes. You should upload this as a separate file labeled 'Manuscript'.

If you would like to make changes to your financial disclosure, please include your updated statement in your cover letter. Guidelines for resubmitting your figure files are available below the reviewer comments at the end of this letter.

If applicable, we recommend that you deposit your laboratory protocols in protocols.io to enhance the reproducibility of your results. Protocols.io assigns your protocol its own identifier (DOI) so that it can be cited independently in the future. For instructions see: http://journals.plos.org/plosone/s/submission-guidelines#loc-laboratory-protocols. Additionally, PLOS ONE offers an option for publishing peer-reviewed Lab Protocol articles, which describe protocols hosted on protocols.io. Read more information on sharing protocols at https://plos.org/protocols?utm_medium=editorial-email&utm_source=authorletters&utm_campaign=protocols.

We look forward to receiving your revised manuscript.

Kind regards,

Professor Hafiz T.A. Khan, Ph.D, CStat

Academic Editor

PLOS ONE

Additional Editor Comments:

Please revise the manuscript as per the comments of both reviewers. You need to check all your statistical results. Statistical p value cannot be 0000. In such case please indicate p<.001 which is highly significant.

Journal Requirements:

When submitting your revision, we need you to address these additional requirements.

1. Please ensure that your manuscript meets PLOS ONE's style requirements, including those for file naming. The PLOS ONE style templates can be found at

https://journals.plos.org/plosone/s/file?id=wjVg/PLOSOne_formatting_sample_main_body.pdf and

https://journals.plos.org/plosone/s/file?id=ba62/PLOSOne_formatting_sample_title_authors_affiliations.pdf

2. Please include your tables as part of your main manuscript and remove the individual files. Please note that supplementary tables should be uploaded as separate "supporting information" files.

3. Thank you for including your ethics statement:  "Each regional public health research institute’s ethical clearance committee reviewed and approved the study. Permission letters were obtained from the head of each selected health center for assessment in the center. All exit and key informant interview participants gave verbal informed consent.".   

Please amend your current ethics statement to include the full name of the ethics committee/institutional review board(s) that approved your specific study.

Once you have amended this/these statement(s) in the Methods section of the manuscript, please add the same text to the “Ethics Statement” field of the submission form (via “Edit Submission”).

For additional information about PLOS ONE ethical requirements for human subjects research, please refer to http://journals.plos.org/plosone/s/submission-guidelines#loc-human-subjects-research.

4. Please include additional information regarding the survey or questionnaire used in the study and ensure that you have provided sufficient details that others could replicate the analyses. For instance, if you developed a questionnaire as part of this study and it is not under a copyright more restrictive than CC-BY, please include a copy, in both the original language and English, as Supporting Information. Moreover, please include more details on how the questionnaire was pre-tested, and whether it was validated.

5. When reporting the results of qualitative research, we suggest consulting the COREQ guidelines or other relevant checklists listed by the Equator Network, such as the SRQR, to ensure complete reporting (http://journals.plos.org/plosone/s/submission-guidelines#loc-qualitative-research). In this case, please consider including more information on the number of interviewers, their training and characteristics; and whether bias issues were considered. Moreover, please provide the interview guide used as a Supplementary file.

6. Please correct your reference to "p=0.000" to "p<0.001" or as similarly appropriate, as p values cannot equal zero.

7. Please provide additional details regarding participant consent. In the ethics statement in the Methods and online submission information, please ensure that you have specified how verbal consent was documented and witnessed.

8. We note that you have indicated that data from this study are available upon request. PLOS only allows data to be available upon request if there are legal or ethical restrictions on sharing data publicly. For information on unacceptable data access restrictions, please see http://journals.plos.org/plosone/s/data-availability#loc-unacceptable-data-access-restrictions.

In your revised cover letter, please address the following prompts:

a) If there are ethical or legal restrictions on sharing a de-identified data set, please explain them in detail (e.g., data contain potentially identifying or sensitive patient information) and who has imposed them (e.g., an ethics committee). Please also provide contact information for a data access committee, ethics committee, or other institutional body to which data requests may be sent.

b) If there are no restrictions, please upload the minimal anonymized data set necessary to replicate your study findings as either Supporting Information files or to a stable, public repository and provide us with the relevant URLs, DOIs, or accession numbers. Please see http://www.bmj.com/content/340/bmj.c181.long for guidelines on how to de-identify and prepare clinical data for publication. For a list of acceptable repositories, please see http://journals.plos.org/plosone/s/data-availability#loc-recommended-repositories.

We will update your Data Availability statement on your behalf to reflect the information you provide.

9. Thank you for stating the following in the Competing Interests section:

"The authors declare that they have no financial or personal relationships which may have inappropriately influenced them in writing this article. The authors have declared that they have no competing interests exist."

We note that one or more of the authors are employed by a commercial company: JSI Research & Training Institute, Inc.

9.1. Please provide an amended Funding Statement declaring this commercial affiliation, as well as a statement regarding the Role of Funders in your study. If the funding organization did not play a role in the study design, data collection and analysis, decision to publish, or preparation of the manuscript and only provided financial support in the form of authors' salaries and/or research materials, please review your statements relating to the author contributions, and ensure you have specifically and accurately indicated the role(s) that these authors had in your study. You can update author roles in the Author Contributions section of the online submission form.

Please also include the following statement within your amended Funding Statement.

“The funder provided support in the form of salaries for authors [insert relevant initials], but did not have any additional role in the study design, data collection and analysis, decision to publish, or preparation of the manuscript. The specific roles of these authors are articulated in the ‘author contributions’ section.”

If your commercial affiliation did play a role in your study, please state and explain this role within your updated Funding Statement.

9.2. Please also provide an updated Competing Interests Statement declaring this commercial affiliation along with any other relevant declarations relating to employment, consultancy, patents, products in development, or marketed products, etc.  

Within your Competing Interests Statement, please confirm that this commercial affiliation does not alter your adherence to all PLOS ONE policies on sharing data and materials by including the following statement: "This does not alter our adherence to  PLOS ONE policies on sharing data and materials.” (as detailed online in our guide for authors http://journals.plos.org/plosone/s/competing-interests) . If this adherence statement is not accurate and  there are restrictions on sharing of data and/or materials, please state these. Please note that we cannot proceed with consideration of your article until this information has been declared.

Please include both an updated Funding Statement and Competing Interests Statement in your cover letter. We will change the online submission form on your behalf.

[Note: HTML markup is below. Please do not edit.]

Reviewers' comments:

Reviewer's Responses to Questions

**Comments to the Author**

1. Is the manuscript technically sound, and do the data support the conclusions?

The manuscript must describe a technically sound piece of scientific research with data that supports the conclusions. Experiments must have been conducted rigorously, with appropriate controls, replication, and sample sizes. The conclusions must be drawn appropriately based on the data presented. 

Reviewer #1: Partly

Reviewer #2: Yes

**********

2. Has the statistical analysis been performed appropriately and rigorously? 

Reviewer #1: Yes

Reviewer #2: Yes

**********

3. Have the authors made all data underlying the findings in their manuscript fully available?

The PLOS Data policy requires authors to make all data underlying the findings described in their manuscript fully available without restriction, with rare exception (please refer to the Data Availability Statement in the manuscript PDF file). The data should be provided as part of the manuscript or its supporting information, or deposited to a public repository. For example, in addition to summary statistics, the data points behind means, medians and variance measures should be available. If there are restrictions on publicly sharing data—e.g. participant privacy or use of data from a third party—those must be specified.

Reviewer #1: Yes

Reviewer #2: Yes

**********

4. Is the manuscript presented in an intelligible fashion and written in standard English?

PLOS ONE does not copyedit accepted manuscripts, so the language in submitted articles must be clear, correct, and unambiguous. Any typographical or grammatical errors should be corrected at revision, so please note any specific errors here.

Reviewer #1: Yes

Reviewer #2: Yes

**********

5. Review Comments to the Author

Please use the space provided to explain your answers to the questions above. You may also include additional comments for the author, including concerns about dual publication, research ethics, or publication ethics. (Please upload your review as an attachment if it exceeds 20,000 characters)

Reviewer #1: I have attached the manuscript with tracked comments. Key issues include;

1. A table with biographic data of participants is essential.

2. You refer to 'quotas of participants' in your sampling. How was this decided.

3. It is unclear if qualitative data was collected using local languages? If this is the case, clarity is needed on how translation was undertaken.

4. Interviews. Were these face to face interviews? Were they audio recorded? If so, were they recorded in different languages? If so, was data translated to English before analysis?

5. Clarity is needed on how many participants took part in; 1. exit interviews; 2. Survey; 3. Key informant interviews.

6. Data analysis - You state that thematic data analysis of qualitative data was undertaken, but no themes were presented. What framework was used for thematic analysis? Who undertook the thematic analysis?

7. The discussion need to be structured around the finds for each research question. The discussion reads like further reporting of results. This needs to focus on what the results mean for the participants and population under study.

8.The implications of the findings need a more rigorous critique. 1. What are the implications of people not renewing their insurance from a health point of view? 2. You report that there is no statistical difference between the quality of service in CBHI and non-CBHI areas. What are the implications of this on the USAID Transform: Primary Health Care project if the project is not making a measurable difference?

Reviewer #2: 1. The objectives are relevant to the study; however, they are broad and need to be revised to make them more specific. Try linking the objective to the overall aim and or the research title to avoid the possibility of answering yes or no.

2. Donabedian model used for the study seems appropriate but the reason for the model selection is not to justify. Make a little comment about other available potential models

3. The use of a mixed-method approach is critical for the study. The sampling technique for the 2 components of the study needs to be more refine and explicit.

4. The method used to recruit participants for the qualitative study should be clearly stated

5. The findings were very important and the conclusions were well written. However, the conclusion can be summary

**********

6. PLOS authors have the option to publish the peer review history of their article (what does this mean?). If published, this will include your full peer review and any attached files.

If you choose “no”, your identity will remain anonymous but your review may still be made public.

**Do you want your identity to be public for this peer review?** For information about this choice, including consent withdrawal, please see our Privacy Policy.

Reviewer #1: **Yes: **Professor K Mafuba, PhD

Reviewer #2: **Yes: **Abdulsalam Ahmed

[NOTE: If reviewer comments were submitted as an attachment file, they will be attached to this email and accessible via the submission site. Please log into your account, locate the manuscript record, and check for the action link "View Attachments". If this link does not appear, there are no attachment files.]

While revising your submission, please upload your figure files to the Preflight Analysis and Conversion Engine (PACE) digital diagnostic tool, https://pacev2.apexcovantage.com/. PACE helps ensure that figures meet PLOS requirements. To use PACE, you must first register as a user. Registration is free. Then, login and navigate to the UPLOAD tab, where you will find detailed instructions on how to use the tool. If you encounter any issues or have any questions when using PACE, please email PLOS at figures@plos.org. Please note that Supporting Information files do not need this step.

Attachment Submitted filename: PONE-D-21-11751_reviewer.pdf Click here for additional data file.

# Author comment: Author response to Decision Letter 0

**DOI:** 10.1371/journal.pone.0256132.r002

2 Jul 2021

Additional Editor Comments:

 Please revise the manuscript as per the comments of both reviewers. You need to check all your statistical results. Statistical p value cannot be 0000. In such case please indicate p<.001 which is highly significant.

 Response: Thank you for your valuable feedback. We have revised the manuscript as per your suggestion.

Journal Requirements:

When submitting your revision, we need you to address these additional requirements.

1. Please ensure that your manuscript meets PLOS ONE's style requirements, including those for file naming. 

 Response: we have checked and attest that all formatting and style requirements have been met. 

2. Please include your tables as part of your main manuscript and remove the individual files. Please note that supplementary tables should be uploaded as separate "supporting information" files.

 Response: we have included the tables as part of the main body of the manuscript and uploaded our additional supplementary information (Supporting Information-Analysis) to provide a full information about data analysis output. 

3. Thank you for including your ethics statement: "Each regional public health research institute’s ethical clearance committee reviewed and approved the study. Permission letters were obtained from the head of each selected health center for assessment in the center. All exit and key informant interview participants gave verbal informed consent." 

Please amend your current ethics statement to include the full name of the ethics committee/institutional review board(s) that approved your specific study.

Once you have amended this/these statement(s) in the Methods section of the manuscript, please add the same text to the “Ethics Statement” field of the submission form (via “Edit Submission”).

For additional information about PLOS ONE ethical requirements for human subjects research, please refer to http://journals.plos.org/plosone/s/submission-guidelines#loc-human-subjects-research.

 Response: We thank the reviewer for the kind words. We have revised the ethical statement in the method section and included the name of the institution which provided ethical approval. The same correction is also made in the submission form.

4. Please include additional information regarding the survey or questionnaire used in the study and ensure that you have provided sufficient details that others could replicate the analyses. For instance, if you developed a questionnaire as part of this study and it is not under a copyright more restrictive than CC-BY, please include a copy, in both the original language and English, as Supporting Information. Moreover, please include more details on how the questionnaire was pre-tested, and whether it was validated.

 Response: Thank you for the suggestion. We included the procedure on questionnaire pretest in the revised manuscript. We also attached the English version of the study questionnaire as a supplement “Supporting Information-Exit interview & facility survey tools”.

5. When reporting the results of qualitative research, we suggest consulting the COREQ guidelines or other relevant checklists listed by the Equator Network, such as the SRQR, to ensure complete reporting (http://journals.plos.org/plosone/s/submission-guidelines#loc-qualitative-research). In this case, please consider including more information on the number of interviewers, their training and characteristics; and whether bias issues were considered. Moreover, please provide the interview guide used as a Supplementary file.

 Response: We considered the suggested COREQ guideline while revising the qualitative analysis section, and we have uploaded our interview guideline as supplementary file to provide clear information about our qualitative data collection (Supplementary file-Interview guide). 

6. Please correct your reference to "p=0.000" to "p<0.001" or as similarly appropriate, as p values cannot equal zero.

 Response: we have revised the statistical values (p-values) in the revised manuscript based on the comments. 

7. Please provide additional details regarding participant consent. In the ethics statement in the Methods and online submission information, please ensure that you have specified how verbal consent was documented and witnessed.

 Response: We have expanded the ‘ethical consideration’ section to reflect how consent was obtained and witnessed. 

8. We note that you have indicated that data from this study are available upon request. PLOS only allows data to be available upon request if there are legal or ethical restrictions on sharing data publicly. For information on unacceptable data access restrictions, please see http://journals.plos.org/plosone/s/data-availability#loc-unacceptable-data-access-restrictions.

 In your revised cover letter, please address the following prompts:

 a) If there are ethical or legal restrictions on sharing a de-identified data set, please explain them in detail (e.g., data contain potentially identifying or sensitive patient information) and who has imposed them (e.g., an ethics committee). Please also provide contact information for a data access committee, ethics committee, or other institutional body to which data requests may be sent.

 b) If there are no restrictions, please upload the minimal anonymized data set necessary to replicate your study findings as either Supporting Information files or to a stable, public repository and provide us with the relevant URLs, DOIs, or accession numbers. Please see http://www.bmj.com/content/340/bmj.c181.long for guidelines on how to de-identify and prepare clinical data for publication. For a list of acceptable repositories, please see http://journals.plos.org/plosone/s/data-availability#loc-recommended-repositories.

 We will update your Data Availability statement on your behalf to reflect the information you provide.

 Response: Thank you, there are no restrictions about data sharing and all relevant data are within the manuscript and we have uploaded the full data set as a supporting file (Study data). we do provide a privilege to update the statement on our behalf. 

9. Thank you for stating the following in the Competing Interests section:

"The authors declare that they have no financial or personal relationships which may have inappropriately influenced them in writing this article. The authors have declared that they have no competing interests exist."

We note that one or more of the authors are employed by a commercial company: JSI Research & Training Institute, Inc.

 9.1. Please provide an amended Funding Statement declaring this commercial affiliation, as well as a statement regarding the Role of Funders in your study. If the funding organization did not play a role in the study design, data collection and analysis, decision to publish, or preparation of the manuscript and only provided financial support in the form of authors' salaries and/or research materials, please review your statements relating to the author contributions, and ensure you have specifically and accurately indicated the role(s) that these authors had in your study. You can update author roles in the Author Contributions section of the online submission form.

Please also include the following statement within your amended Funding Statement.

“The funder provided support in the form of salaries for authors [insert relevant initials], but did not have any additional role in the study design, data collection and analysis, decision to publish, or preparation of the manuscript. The specific roles of these authors are articulated in the ‘author contributions’ section.”

If your commercial affiliation did play a role in your study, please state and explain this role within your updated Funding Statement.

 9.2. Please also provide an updated Competing Interests Statement declaring this commercial affiliation along with any other relevant declarations relating to employment, consultancy, patents, products in development, or marketed products, etc. 

 Within your Competing Interests Statement, please confirm that this commercial affiliation does not alter your adherence to all PLOS ONE policies on sharing data and materials by including the following statement: "This does not alter our adherence to PLOS ONE policies on sharing data and materials.” (as detailed online in our guide for authors http://journals.plos.org/plosone/s/competing-interests) . If this adherence statement is not accurate and there are restrictions on sharing of data and/or materials, please state these. Please note that we cannot proceed with consideration of your article until this information has been declared.

 Please include both an updated Funding Statement and Competing Interests Statement in your cover letter. We will change the online submission form on your behalf.

 Response: Thank you for your suggestion. We have taken all the funding statement and computing interest statement in our cover letter. To make clear we also included author’s contribution in the cover letter. 

*****************************************

Reviewers' comments:

Reviewer's Responses to Questions

Comments to the Author

1. Is the manuscript technically sound, and do the data support the conclusions?

The manuscript must describe a technically sound piece of scientific research with data that supports the conclusions. Experiments must have been conducted rigorously, with appropriate controls, replication, and sample sizes. The conclusions must be drawn appropriately based on the data presented. 

 Reviewer #1: Partly

 Reviewer #2: Yes

________________________________________

2. Has the statistical analysis been performed appropriately and rigorously? 

 Reviewer #1: Yes

 Reviewer #2: Yes

________________________________________

3. Have the authors made all data underlying the findings in their manuscript fully available?

The PLOS Data policy requires authors to make all data underlying the findings described in their manuscript fully available without restriction, with rare exception (please refer to the Data Availability Statement in the manuscript PDF file). The data should be provided as part of the manuscript or its supporting information, or deposited to a public repository. For example, in addition to summary statistics, the data points behind means, medians and variance measures should be available. If there are restrictions on publicly sharing data—e.g. participant privacy or use of data from a third party—those must be specified.

 Reviewer #1: Yes

 Reviewer #2: Yes

________________________________________

4. Is the manuscript presented in an intelligible fashion and written in standard English?

PLOS ONE does not copyedit accepted manuscripts, so the language in submitted articles must be clear, correct, and unambiguous. Any typographical or grammatical errors should be corrected at revision, so please note any specific errors here.

 Reviewer #1: Yes

 Reviewer #2: Yes

________________________________________

*****************************************

5. Review Comments to the Author

Please use the space provided to explain your answers to the questions above. You may also include additional comments for the author, including concerns about dual publication, research ethics, or publication ethics. (Please upload your review as an attachment if it exceeds 20,000 characters)

Reviewer #1: I have attached the manuscript with tracked comments. Key issues include.

1. A table with biographic data of participants is essential.

Response: Thank you for the suggestion. We have added a table to display the participants characteristics per individual CBHI enrollment status. 

2. You refer to 'quotas of participants' in your sampling. How was this decided?.

Response: Thank you for your question. We followed a consecutive sampling until we get adequate sample from each sampled Health centers. We have revised statements to clearly present how many participants were included from health facilities in CBHI and non-CBHI implementing woredas. 

3. It is unclear if qualitative data was collected using local languages? If this is the case, clarity is needed on how translation was undertaken.

Response: Qualitative data was collected using key informant interview guide. All interviews were done using local languages. The tools in English language were translated to local language and then back to English to make sure the concepts are not lost in the translation process. We have revised statements to clearly present how the data collected and analyzed 

4. Interviews. Were these face to face interviews? Were they audio recorded? If so, were they recorded in different languages? If so, was data translated to English before analysis?

Response: Yes, both the qualitative and quantitative data was collected using local languages, and then back translated into English before analysis. The key informants’ responses were audio-recorded, transcribed verbatim in local languages (Amharic, Oromifa and Tigrigna) were back translated into English and thematic analysis was used to analyze the data. The data process statement was revised to clearly present qualitative data analysis in the revised version of the manuscript. 

5. Clarity is needed on how many participants took part in; 1. exit interviews; 2. Survey; 3. Key informant interviews.

Response: Thank you for the issues raised, we were considered 560 exit interviews, 56 facility survey and 28 key informant interviews. We have revised statements to clearly present how many participants included, the data collected and analyzed.

6. Data analysis - You state that thematic data analysis of qualitative data was undertaken, but no themes were presented. What framework was used for thematic analysis? Who undertook the thematic analysis?

Response: Thank you, we have revised statements to clearly present how the qualitative data analysis was done and who undertook the analysis, the following statements are included in the main body of the manuscript under data analysis: 

“The key informant participant responses were audio-recorded, transcribed verbatim in local languages (Amharic, Oromifa and Tigrigna) were back translated into English and thematic analysis was used to analyze the data. We had used three phases data analysis: preparation, team organization and reporting the summery result in each team. The first phase of the analysis started from careful reading of the data multiple times to become immersed in and familiar with the data. In the organization phase, each transcript was read carefully by the first author, highlighting the theme text (words or phrases) that appeared to describe the phenomenon under the study (use of CBHI, quality service, its interaction). The highlighted themes texts were openly and manually coded by giving each them text a descriptive code. The third author read the data to confirm the descriptive codes. These codes were revised, and the codes that emerged from the revision were jointly reviewed before integrating them into the analysis. The other authors reviewed with the collaboration of first and third author, discussed, and agreed on the final code categories. The final analysis was summarized manually based on agreed emerging themes”. 

7. The discussion need to be structured around the finds for each research question. The discussion reads like further reporting of results. This needs to focus on what the results mean for the participants and population under study.

Response: Thank you for the suggestion. We have moved part of the discussion, that reads like a result, into the result section. Moreover, we have added a paragraph that introduces the discussion outline which presented interpretation of the findings by triangulating different components of the study as well comparing the findings with related studies, to facilitate understanding of observed nuances that influence CBHI and quality interactions and also identify gaps for future investigation. 

8. The implications of the findings need a more rigorous critique. 1. What are the implications of people not renewing their insurance from a health point of view? 2. You report that there is no statistical difference between the quality of service in CBHI and non-CBHI areas. What are the implications of this on the USAID Transform: Primary Health Care project if the project is not making a measurable difference?

Response: Thank you for the suggestion, the study showed that perceived quality of health care is essential for enrollment and renewal, although we were not able to generate strong evidence that CBHI improved quality of service. This study was not designed to investigate the effect of CBHI enrollment on individuals’ health outcome (morbidity or mortality associated with insurance). However, we assume the collective risk and financial burden sharing due to enrolment will result in better health outcome in the long run. If the people not renewing their insurance, there is no specific harm from the health point of view. They may be required to pay from out-of-pocket next time when they seek health care and it may lead to catastrophic health expenditure. One thing to note here is that choosing to enroll in insurance is a complicated decision, and quality of health service is only one factor that influences this choice. The study recognizes the complexity of linking insurance with quality of health care and mentioned as a limitation of the study and recommends further follow-up research to look at the link between healthcare service quality and CBHI enrollment or renewal. The result does not have any implication on TRANSFORM project, but for ease of management, we conducted the study in transform intervention areas as a study site.

Reviewer #2: 

1. The objectives are relevant to the study; however, they are broad and need to be revised to make them more specific. Try linking the objective to the overall aim and or the research title to avoid the possibility of answering yes or no.

 Response: Thank you for kind words, addressed accordingly in the revised version

2. Donabedian model used for the study seems appropriate but the reason for the model selection is not to justify. Make a little comment about other available potential models

 Response: We have added a statement explaining that Donabedian model was a good fit for purpose to this assessment because it explores all the three elements of quality (structure, process and outcome) which are interlinked. “This model was adopted because it explores all the three elements of quality and postulates that the CBHI implementation mechanism itself and the related enhanced revenue generation would improve quality of health care service; enhanced quality of health care service or perceived quality itself would generate greater enrollment into CBHI”. 

3. The use of a mixed-method approach is critical for the study. The sampling technique for the 2 components of the study needs to be more refine and explicit.

 Response: Thank you for your feedback. We have revised statements to clearly present how the sample and sampling technique used.

4. The method used to recruit participants for the qualitative study should be clearly stated

Response: Thank you, the revision was made in the manuscript 

5. The findings were very important, and the conclusions were well written. However, the conclusion can be summary

Response: Thank you. We addressed it accordingly.

Attachment Submitted filename: point by point Response to Reviewers_f.docx Click here for additional data file.

# Peer review: Decision Letter 1

**DOI:** 10.1371/journal.pone.0256132.r003

**Authors:** Hafiz T.A. Khan

30 Jul 2021

The interaction of healthcare service quality and community-based health insurance in Ethiopia

PONE-D-21-11751R1

Dear Authors,

We’re pleased to inform you that your manuscript has been judged scientifically suitable for publication and will be formally accepted for publication once it meets all outstanding technical requirements.

Within one week, you’ll receive an e-mail detailing the required amendments. When these have been addressed, you’ll receive a formal acceptance letter and your manuscript will be scheduled for publication.

An invoice for payment will follow shortly after the formal acceptance. To ensure an efficient process, please log into Editorial Manager at http://www.editorialmanager.com/pone/, click the 'Update My Information' link at the top of the page, and double check that your user information is up-to-date. If you have any billing related questions, please contact our Author Billing department directly at authorbilling@plos.org.

If your institution or institutions have a press office, please notify them about your upcoming paper to help maximize its impact. If they’ll be preparing press materials, please inform our press team as soon as possible -- no later than 48 hours after receiving the formal acceptance. Your manuscript will remain under strict press embargo until 2 pm Eastern Time on the date of publication. For more information, please contact onepress@plos.org.

Kind regards,

Professor Hafiz T.A. Khan, Ph.D, CStat

Academic Editor

PLOS ONE

Additional Editor Comments (optional):

Reviewers' comments:

Reviewer's Responses to Questions

**Comments to the Author**

1. If the authors have adequately addressed your comments raised in a previous round of review and you feel that this manuscript is now acceptable for publication, you may indicate that here to bypass the “Comments to the Author” section, enter your conflict of interest statement in the “Confidential to Editor” section, and submit your "Accept" recommendation.

Reviewer #1: All comments have been addressed

Reviewer #2: All comments have been addressed

**********

2. Is the manuscript technically sound, and do the data support the conclusions?

The manuscript must describe a technically sound piece of scientific research with data that supports the conclusions. Experiments must have been conducted rigorously, with appropriate controls, replication, and sample sizes. The conclusions must be drawn appropriately based on the data presented. 

Reviewer #1: Yes

Reviewer #2: Yes

**********

3. Has the statistical analysis been performed appropriately and rigorously? 

Reviewer #1: Yes

Reviewer #2: Yes

**********

4. Have the authors made all data underlying the findings in their manuscript fully available?

The PLOS Data policy requires authors to make all data underlying the findings described in their manuscript fully available without restriction, with rare exception (please refer to the Data Availability Statement in the manuscript PDF file). The data should be provided as part of the manuscript or its supporting information, or deposited to a public repository. For example, in addition to summary statistics, the data points behind means, medians and variance measures should be available. If there are restrictions on publicly sharing data—e.g. participant privacy or use of data from a third party—those must be specified.

Reviewer #1: Yes

Reviewer #2: Yes

**********

5. Is the manuscript presented in an intelligible fashion and written in standard English?

PLOS ONE does not copyedit accepted manuscripts, so the language in submitted articles must be clear, correct, and unambiguous. Any typographical or grammatical errors should be corrected at revision, so please note any specific errors here.

Reviewer #1: Yes

Reviewer #2: Yes

**********

6. Review Comments to the Author

Please use the space provided to explain your answers to the questions above. You may also include additional comments for the author, including concerns about dual publication, research ethics, or publication ethics. (Please upload your review as an attachment if it exceeds 20,000 characters)

Reviewer #1: Thank you for addressing my questions. I have no further comments. I enjoyed reading your paper and wish all the best with the publication.

Reviewer #2: All the raised concerned are appropriately addressed and the article has been considered for publication without further review.

**********

7. PLOS authors have the option to publish the peer review history of their article (what does this mean?). If published, this will include your full peer review and any attached files.

If you choose “no”, your identity will remain anonymous but your review may still be made public.

**Do you want your identity to be public for this peer review?** For information about this choice, including consent withdrawal, please see our Privacy Policy.

Reviewer #1: **Yes: **Professor Kay Mafuba

Reviewer #2: **Yes: **Abdulsalam Ahmed

# Editor report: Acceptance letter

**DOI:** 10.1371/journal.pone.0256132.r004

**Authors:** Hafiz T.A. Khan

11 Aug 2021

PONE-D-21-11751R1 

The interaction of healthcare service quality and community-based health insurance in Ethiopia 

Dear Dr. Tefera:

I'm pleased to inform you that your manuscript has been deemed suitable for publication in PLOS ONE. Congratulations! Your manuscript is now with our production department. 

If your institution or institutions have a press office, please let them know about your upcoming paper now to help maximize its impact. If they'll be preparing press materials, please inform our press team within the next 48 hours. Your manuscript will remain under strict press embargo until 2 pm Eastern Time on the date of publication. For more information please contact onepress@plos.org.

If we can help with anything else, please email us at plosone@plos.org. 

Thank you for submitting your work to PLOS ONE and supporting open access. 

Kind regards, 

PLOS ONE Editorial Office Staff

on behalf of

Professor Hafiz T.A. Khan 

Academic Editor

PLOS ONE
